# Effects of temperature on the life-history traits of *Myzus persicae* and its efficiency in transmitting potato virus Y (PVY) in potato crops

**DOI:** 10.7717/peerj.21239

**Published:** 2026-06-04

**Authors:** Bonoukpoè M. Sokame, Henri E.Z. Tonnang, Heidy Gamarra, Pablo Carhuapoma, Jan Kreuze, Leah R. Johnson, Ali Arab, Peter A. Armbruster, Oswaldo C. Villena

**Affiliations:** 1 International Centre of Insect Physiology and Ecology (ICIPE), Nairobi, Kenya; 2 International Institute of Tropical Agriculture - IITA, Ibadan, Nigeria; 3 School of Agriculture, Earth, and Environmental Sciences, University of KwaZulu-Natal, Pietermaritzburg, South Africa; 4 International Potato Center - CIP, Lima, Peru; 5 Department of Statistics, Virginia Tech, Blacksburg, VA, United States of America; 6 Department of Mathematics and Statistics, Georgetown University, Washington, DC, United States of America; 7 Department of Biology, Georgetown University, Washington, DC, United States of America; 8 The Earth Commons Institute, Georgetown University, Washington, DC, United States of America

**Keywords:** Global warming, Aphids, Virus transmission, Staple crops, Food security

## Abstract

Aphids are highly sensitive to temperature changes and play a crucial role in transmitting plant viruses, accounting for the transmission of more than 50% of viruses that cause disease in crops. Among them, *Myzus persicae* is a major global pest, affecting over 400 plant species and transmitting more than 100 plant viruses, including potato virus Y (PVY), which poses a severe threat to potato crops. This study examines how temperature influences the life-history traits of *M. persicae* and its efficiency in transmitting PVY. Our research revealed that temperature significantly affects developmental duration, survival, and fecundity of *M. persicae*. The aphids exhibited the longest lifespan at 10 °C and the shortest at 30 °C. Similarly, fecundity declined from 29.81 offspring per female at 10 °C to 14.25 at 30 °C. PVY transmission efficiency was highest at 20 °C. We also mapped potential PVY transmission regions and identified tropical and subtropical areas as high-risk due to their favourable temperatures and predicted abundance of aphids. Regional temperature differences significantly influence aphid development and PVY spread, necessitating localized management strategies. Our findings emphasize the importance of integrating climatological, ecological, and epidemiological data to develop robust pest/disease management strategies that mitigate the impact of *M. persicae* and PVY on potato production, thereby enhancing global food security.

## Introduction

The green peach aphid, *Myzus persicae* Sulzer (Hemiptera: Aphididae), is a globally significant pest that affects a wide range of crops due to its ability to feed on over 400 plant species including potato plants (*Solanum tuberosum* L.) and transmit more than 100 plant viruses including Potato virus Y (PVY) (Blackman & Eastop, 2000). *Myzus persicae* causes direct damage to potato plants through its feeding activity. Aphids feed by inserting their stylets into the phloem of the plant and extracting sap, which weakens the plant, and can lead to symptoms such as chlorosis, leaf curling, and reduced growth rates (Radcliffe & Ragsdale, 2002). Heavy infestations can cause significant reductions in photosynthesis and overall plant vigour, ultimately impacting tuber yield and quality. Yield losses of up to 50% in potato crops have been reported (Kroschel et al., 2020). However, the more insidious damage comes from the transmission of plant viruses such as PVY. *Myzus persicae* is the most efficient vector of PVY, spreading the virus as it feeds on potato plants (Radcliffe & Ragsdale, 2002; Boquel, Ameline & Giordanengo, 2011). Less efficient vectors are *Macrosiphum euphorbiae* and *Sitobion avenae* (Boquel, Ameline & Giordanengo, 2011). PVY is one of the most economically damaging viruses affecting potatoes. It belongs to the *Potyviridae* family and is responsible for causing a range of symptoms including mottling, leaf drop, and tuber necrosis, which significantly diminish the marketability and yield of potato crops (Singh et al., 2008). Infected plants often produce fewer and smaller tubers, and tubers may have necrotic rings, making them unmarketable. Estimates suggest that PVY can reduce potato yields by up to 80% in severely affected fields (Karasev & Gray, 2013). The economic losses are compounded by the increased costs associated with managing the disease and the reduced marketability of infected potatoes. Understanding the dynamics of *M. persicae* infestations and PVY transmission is critical for developing effective management strategies to protect potato production.

To mitigate the impact of *M. persicae* and PVY, integrated pest management (IPM) strategies are essential. These strategies include the use of certified virus-free seed potatoes, crop rotation, and the timely application of insecticides to control aphid populations (Radcliffe, Hutchinson & Cancelado, 2009). Biological control agents, such as natural predators and parasitoids, also reduce aphid numbers. Additionally, the deployment of PVY-resistant potato varieties is effective in reducing the incidence of the disease (Singh et al., 2008). Despite advances in IPM, challenges remain in managing *M. persicae* and PVY due to factors such as insecticide resistance, the emergence of new PVY strains, the rapid non- circulative transmission mode of PVY, and climatic conditions (Brault et al., 2010; Whitfield, Falk & Rotenberg, 2015). Therefore, understanding the ecological and epidemiological interactions between aphids and PVY under changing environmental conditions remains a high priority. The study of *M. persicae*’s life history traits and its efficiency in transmitting PVY under varying temperature conditions is crucial for developing effective pest management strategies.

Temperature is a critical environmental factor that influences the biology, behaviour, and ecology of insects. For *M. persicae*, temperature affects developmental rates, survival, fecundity, and virus transmission efficiency (Régnière, St-Amant & Duval, 2012; Li et al., 2025). Understanding these temperature-dependent life history traits is essential for predicting aphid population dynamics and virus spread under changing climatic conditions, which in turn can inform IPM practices (Jha et al., 2024; Scherm, 2004). Numerous studies have explored the influence of temperature on aphid biology. For instance, Khurshid et al. (2022) demonstrated that the developmental rates of *M. persicae* increase with temperature up to an optimal point, beyond which higher temperatures become detrimental to survival and reproduction. This aligns with the findings of Baral et al. (2022), who demonstrated a similar trend in the development rates of *M. persicae* with increasing temperature. Additionally, Jeger et al. (2004) reported that temperature variations significantly impact the transmission efficiency of plant viruses by aphids, underscoring the need for temperature-specific management strategies in pest control. Although previous studies (*e.g.*, Baral et al. 2022; Khurshid et al. 2022) have reported the influence of temperature on *M. persicae* biology, these have largely focused on populations outside Africa and Latin America (European or Asian populations). Yet, aphid populations show regional genetic divergence and life-cycle plasticity (Blackman, 1974; Margaritopoulos et al., 2002), which may influence their demographic responses and virus transmission potential. To date, no detailed life table studies have been conducted on East African or Peruvian *M. persicae* populations. Furthermore, there is a need for comprehensive studies that integrate the effects of temperature on both the life history traits of *M. persicae* and its ability to transmit PVY. Such integrated assessments are vital for developing robust predictive models and effective management practices, particularly in the context of global climate change (Intergovernmental Panel on Climate Change (IPCC), 2015).

This study aims to address this gap by systematically investigating the influence of a range of temperatures on the survival, development, fecundity, and PVY transmission rates of *M. persicae*. We hypothesize that temperature significantly affects these parameters and that there is an optimal temperature range where *M. persicae* exhibits peak performance in terms of survival and reproduction, while extreme temperatures (both high and low) adversely affect these traits. Additionally, we anticipate that the efficiency of PVY transmission by *M. persicae* will exhibit a temperature-dependent pattern, potentially complicating disease management under fluctuating climatic conditions.

## Materials and Methods

### *Myzus persicae* life-history traits

#### Plant material and aphid colonies

Portions of this text were previously published as part of a preprint (Sokame et al., 2024). For generating *M. persicae* life table data, a colony of *M. persicae* originating from potato crops in Kenya and maintained on potato plants for five years in a greenhouse facility at the International Centre of Insect Physiology and Ecology (*icipe*) was used for the experiments. The rearing conditions were controlled at 20–23 °C, 70–95% RH, and a 12:12 h light-dark photoperiod. The potato variety “Shangi,” known for its popularity and moderately high yields in Kenya, served as the host plant. Leaves from pesticide-free plants at the vegetative stage were used throughout the experiment. Before use, the leaves were disinfected in a 1% sodium hypochlorite solution for 5 min, followed by thorough rinsing with tap and distilled water (Gao et al., 2012; Liu et al., 2021). This procedure ensured high-quality leaves for the aphids. During the experiments, leaf discs showing signs of chlorosis or dehydration were replaced, and the insects were carefully transferred to fresh discs using a paintbrush.

#### Development and survival

Individual females of aphid *M. persicae* were transferred to separate Petri dishes (10 cm diameter) containing a host plant leaf disc and 1% agar solution. The dishes were kept at 22 ± 1 °C and 70 ± 10% relative humidity for 6 h; after that the females and all the nymphs, except for ten per dish, were removed. The dishes with newly hatched neonates were then incubated in environmental chambers (phytotron; Weiss Technik, Heuchelheim, Germany) at different constant temperatures 10, 15, 20, 25, and 30 °C at 70 ± 10% relative humidity and a 12-hour photoperiod. Juvenile development and survival were monitored daily until they developed into adult aphids, the females of which were then used in the fecundity experiment described below (Gao et al., 2012; Liu et al., 2021). Ten replicates of a cohort of 10 nymphs per Petri dish were tested for each temperature.

#### Adult longevity and fecundity

A simple random sampling design was used, which included five temperature variables (10, 15, 20, 25, and 30 °C) and 100 repetitions. Female adult aphids were incubated at the appropriate temperature in dishes containing potato host plant leaf discs, maintained under a 12-hour photoperiod, and were transferred to new leaf discs when necessary (Liu et al., 2021). The pre-reproductive and reproductive periods were evaluated under a stereomicroscope every 24 h, and the number of nymphs produced, and their longevity was determined at each temperature. All nymphs produced were removed to eliminate the potential effect of crowding.

#### Key population parameters and formulas

There are a variety of demographic summaries that capture multiple important aspects of population growth. Many of these are related to each other and are usually calculated together. Here we summarize the population metrics calculated in this study following Gao et al. (2012). The first four are the key life-history/demographic parameters on which the other metrics are built:

(1) Age-stage specific survival rate (*Sxj*) represents the probability that an individual will survive to age *x* and stage *j*.

(1) \begin{eqnarray*}{S}_{xj}= \frac{{n}_{xj}}{{n}_{01}} \end{eqnarray*}

where *n*_xj_ is the number of individuals surviving to age x and stage j, and *n*_01_ is the initial number of neonates.

(2) Age-specific survival rate *(lx)* indicates the probability that a newly laid neonate will survive to age x.

(2) \begin{eqnarray*}{\mathrm{l}}_{x}=\sum _{j=1}^{k}{S}_{xj}\end{eqnarray*}

where *k* is the number of developmental stages.

(3) Age-specific fecundity *(mx)* represents the mean number of offspring produced per individual at age x.

(3) \begin{eqnarray*}{m}_{x}= \frac{\sum _{j=1}^{k}{S}_{xj}{f}_{xj}}{\sum _{j=1}^{k}{S}_{xj}} \end{eqnarray*}

where *f*_*xj*_ is the fecundity of individuals at age x and stage j.

(4) Age-stage life expectancy (e_*xj*_) indicates the expected lifespan of individuals at age *x* and stage *j*.

(4) \begin{eqnarray*}{e}_{xj}=\sum _{i=x}^{n}\sum _{y=j}^{m}{\mathrm{S}}_{ij}^{{^{\prime}}}\end{eqnarray*}

where ${S}_{\mathrm{ij}}^{{^{\prime}}}$ is the probability that an individual of age *x* and stage *j* will survive to age *i* and stage *y*.

There are multiple summaries of population growth that can be built from the four demographic parameters above. Because these capture different aspects of population performance we choose to include the following five:

(1) Net reproductive rate *(R*_0_) estimates the total number of offspring that an individual is expected to produce over its lifetime and is calculated as:
  (5) \begin{eqnarray*}{\mathrm{R}}_{0}=\sum _{x=0}^{\infty }{l}_{x}{m}_{x}\end{eqnarray*}
(2) Intrinsic rate of increase (r_*m*_) is calculated using the Euler-Lotka equation, representing the growth rate of the population.
  (6) \begin{eqnarray*}\sum _{x=0}^{\infty }x{e}^{-x{r}_{m}}{l}_{x}{m}_{x}=1\end{eqnarray*}
(3) Finite rate of increase (*λ*) indicates the population’s growth rate per unit time.
  (7) \begin{eqnarray*}\lambda ={e}^{{r}_{m}}\end{eqnarray*}
(4) Mean generation time *(T)* represents the average time between the birth of an individual and the birth of its offspring and is calculated as follow:

(8) \begin{eqnarray*}T= \frac{ln{R}_{0}}{{r}_{m}} \end{eqnarray*}

(5) Doubling time *(DT)* is a key demographic parameter used to describe the period required for a population to double in size. The concept of doubling time is based on exponential growth, where the population increases at a constant rate over time.

(9) \begin{eqnarray*}DT= \frac{ln(2)}{{r}_{m}} .\end{eqnarray*}

#### Data analysis

Count data (nymph longevity, adult longevity, total longevity, and fecundity expressed as offspring per female) and life table parameters data (net reproduction rate (*R*_*o*_), intrinsic rate of increase (*r*_*m*_), finite rate of increase (*λ*), mean generation time (*T*), doubling time (*DT*)) were tested for normality using Shapiro–Wilk test and homogeneity of variance using Levene test. The data were not normally distributed, and variances were not homogeneous. Count data were therefore, analysed with generalized linear model (GLM) with negative binomial error distribution considering overdispersion. Whenever there was a significant difference, the means were separated using Tukey’s honest significant difference (HSD) test using “*agricolae*” package in R (de Mendiburu, 2023; R Core Team, 2021). Life table parameter data were analysed using Kruskal-Wallis nonparametric procedure. The survival and life expectancy curves for immature life stage and adult of *M. percicae* were generated using Kaplan–Meier estimator method, and log-rank test was used to compare the effect of temperature on *M. persicae* immature and adult in SigmaPlot software version 14.0 (Systat Software, Inc., San Jose, CA, USA).

### *Myzus persicae* phenology model development and analysis

The development of the *M. persicae* phenology model simulation was conducted using the Insect Life Cycle Modelling (ILCYM) software version 4.0, developed by the International Potato Center (CIP) (Sporleder et al., 2020). ILCYM is a software that facilitates the development of pest insect phenology models and provides analytical tools for studying pest population ecology. The ILCYM software, which employs R statistics (R Core Team, 2021) for all calculations, is freely available online. Development time, development rate, senescence, mortality, and total oviposition data collected under different constant temperature conditions were included in the phenology model. The best-fit model was selected based on the Akaike Information Criterion (AIC), with lower AIC values indicating better fit (Akaike, 1998; Sporleder et al., 2017). Data were transformed into interval censored time-to-event data for survival analysis. Parametric accelerated failure time (AFT) modelling was used to determine medians and the distribution of development times, with models adjusted using the *survreg* procedure in R (Kalbfleisch & Prentice, 2002; Therneau, 2020).

For development times and adult longevity, log-error distributions were assumed, and the most appropriate distribution link function (log–logistic, lognormal, or Weibull) was chosen based on maximum likelihood. These functions were fitted in terms of ln-times. Lower developmental thresholds and thermal requirements were calculated using linear regression between temperature and observed development rates within the linear range. Survival time of immature stages was calculated from the relative frequency of surviving insects, and different nonlinear models were adjusted by regression to describe mortality rates and fecundity by temperature. Development rate was expressed as the reciprocal of mean development times, and mortality was calculated from cohort mortality frequency.

### Transmission rates of potato virus Y (PVY) by *Myzus persicae*

#### Plant material, aphid colony, and PVY strain

In this study, potato (*Solanum tuberosum* cv. “Yungay”) was selected as the host plant. These were virus-free potato plants obtained from the CIP germplasm bank (Vollmer et al., 2022). The *M. persicae* aphids used in the study came from a long-standing colony reared at CIP’s insectary for six years. Aphid colonies are maintained at 20–23 °C, with over 70% relative humidity, and 12:12 (L:D) photoperiod (Gamarra et al., 2020). The PVY isolate used in this study was obtained from the CIP virus collection; it was the PVY-O strain and was confirmed by reverse transcription polymerase chain reaction (RT-PCR) (Amao & Muller, 2025). This strain corresponds to the sequence Potato virus Y isolate NMG-7, accession number: MN607725, available in NCBI. This strain was propagated in potato plants, and it was chosen because of its global distribution, high virulence, and significant impact on potato production, making it a relevant model for epidemiological and management studies. Virus-infected tubers, confirmed by RT-PCR 25 days after planting, served as the source plants for the transmission tests (Gamarra et al., 2020). Healthy sprouted potatoes, prepared for inoculation, were planted seven days before the trials and maintained under the same conditions as the virus transmission experiments, at temperatures of 12 °C, 15 °C, 20 °C, and 25 °C.

#### Virus transmission experiments

Transmission of PVY-O by *M. persicae* was assessed at four different temperatures of 12 °C, 15 °C, 20 °C, and 25 °C. We exposed the insects to temperatures where transmission is successful, according to references that studied the effects of temperature on the acquisition and transmission of PVY. The literature reports that the optimal concentration of PVY is maintained within a temperature range of 15 to 25 °C, as higher temperatures affect the stability of the virus. Furthermore, at temperatures below 10 °C or above 25 °C, potato crop development can be negatively affected, which indirectly influences the concentration and spread of the virus (Chung et al., 2016). Three replications were performed for each temperature. To conduct the experiment, 30 aphids were placed on infected potato plants, which were arranged in boxes covered with a fine mesh netting and placed in environmental chambers set to one of the four temperatures (Gamarra et al., 2020). Aphids were restricted to two specific leaves on each plant: one leaf from the top and one from the middle. Five aphids were placed on each leaf by plant and isolated from the others using clip-cages (17 × 13 × 13 cm). Each group of 10 aphids feeding on a single plant represented one replication. The aphids were allowed to feed for 5 min during the acquisition access period (AAP), after being fasted for two hours (Gamarra et al., 2020).

Following the AAP, each individual aphid was transferred to virus-free potato sprouts (10 days old), where aphids were allowed to feed for a 24-hour inoculation access period (IAP). After the 24-hour IAP, the aphids were removed from potato plants, and a 0.1% solution of Spirotetramat insecticide was applied to ensure no insects remained. The potato plants were then placed in a greenhouse for 25 days at a temperature of 18 °C and a photoperiod of 12:12 (L:D). After this period, non-inoculated leaves from the plants were collected and tested for the presence of PVY using RT-PCR analysis (Fig. S1) (Gamarra et al., 2020).

#### Reverse transcription polymerase chain reaction (RT-PCR)

Total RNA was extracted from potato leaf samples using the Cetyltrimethylammonium bromide (CTAB) method adapted from Lodhi et al. (1994). Ensuring thorough mixing and precipitation to maximize yield. For cDNA synthesis, a mixture of 10 µl of nuclease-free water (NFW), 1 µl of Random primers (250 ng/µl), and 1 µl of total RNA (500 ng/µl) was prepared and denatured at 65 °C for 10 min in a thermocycler (Applied Biosystems, Foster City, CA, USA), then cooled to 10 °C for 5 min. Next, an RT Mix containing 4 µl 5X First Strand Buffer, 0.5 µl dNTPs (10 mM), 0.5 µl DTT (100 mM), 0.5 µl RNAse OUT, and 0.5 µl M-MLV reversed transcriptase was added. The mixture was then incubated at 37 °C for 50 min, heated at 95 °C for 15 min, and cooled to 10 °C, resulting in a cDNA volume of 20 µl, which was then diluted with 40 µl of NFW for PCR analysis (Gamarra et al., 2020). PVY detection was performed using RT-PCR primers targeting the coat protein (CP) gene: forward primer PVY-F (5′-AAA TAC TCG RGC AAC TCA ATC ACA G-3′) and reverse primer PVY-R (5′-CGC TTC TGC AAC ATC TGA AAT GT-3′), which amplify a 265 bp fragment. These primers have been designed at CIP (Amao & Muller, 2025).

PCR conditions were as described by Singh et al. (2008). Amplicons were visualized on 1% agarose gels stained with GelRed. The reaction mixture included 4 µL of PCR 5X Buffer (Promega), 1 µl of MgCl2 (25 mM), 0.5 µl of dNTPs (10 mM), 0.5 µL of primers (PVY_F/PVY_R) at 10 µM each, 0.125 µl of the Taq polymerase, 8.375 µl NFW, and 5 µl of diluted cDNA. The mixture was initially heated to 95 °C for 2 min, followed by 35 cycles at a temperature of 94 °C for 30 s, 57 °C for 45 s, and 72 °C for 45 s, concluding with a final extension at 72 °C for 10 min. The PCR products were then visualised on a 1% agarose gel stained with Gelred (Invitrogen) under ultraviolet light. To ensure reliability, negative and positive controls were included, and reactions were performed in replicates (Gamarra et al., 2020).

#### Developing the mathematical model for virus transmission efficiency

For each adult in a n_j_ sample at a specific temperature (*T*_*j*_), the binary variable y_ij_ indicated transmission success (1) or failure (0) for the ith insect at temperature *T*_*j*_. This method allowed us to calculate the proportion of insects transmitting the virus at each temperature, resulting in a transmission rate where temperature served as the independent variable and transmission percentage as the dependent variable, described by the non-linear function *f(T)*=* p*. We evaluated 20 ILCYM insect transmission rate models, selecting the optimal ones based on the corrected Akaike Information Criterion (AICc). The Levenberg–Marquardt algorithm in ILCYM’s (More, 1978) estimated model parameters by minimizing the residual sum of squares (*via* R’s *minpack.lm* package). After assessing several models, the Taylor model was chosen for its strong fit (Table S6). This model was then integrated into the insect phenology analysis (previously processed in ILCYM) as an alternative approach for studying temperature-dependent transmission in insect vectors (Sporleder et al., 2017; Gamarra et al., 2020; Sporleder et al., 2023). All statistical computations and model implementation were performed in R version 3.4.1 (R Core Team, 2021; Gamarra et al., 2020).

### Risk index mapping

To implement and assess the risk of *M. persicae* using its phenological model in a GIS (Geographic Information System) environment, we used the potential distribution and risk mapping module of the ILCYM 4.0 software, which enables spatial simulations at regional and global scales. This approach makes it possible to map the vulnerability of geographic areas to the potential presence of this pest, estimating both its likelihood of establishment and its possible abundance, following the methodology described by Sporleder et al. (2017; 2023) and Kroschel et al. (Kroschel et al., 2013; Kroschel, Sporleder & Carhuapoma, 2016). The risk of establishment was estimated using the Establishment Index (EI), while potential abundance was assessed using the Generation Index (GI), focusing on regions where potatoes are cultivated worldwide.

### Transmission model validation

To evaluate the model based on presence-absence data, it is first necessary to establish a threshold value. This critical point distinguishes “presence” (values above the threshold) from “absence” (values below it). Consequently, sites with high predicted suitability values (VTI > critical point) are expected to correspond to known presence areas, which can be compared with the recorded presence coordinates of PVY. Conversely, locations with lower predicted suitability values tend to be areas where the presence of the species is unknown (absence/pseudo-absence). Following this, the True Positive Rate (sensitivity) and False Positive Rate (1-specificity) can be determined. This evaluation is summarized by calculating the total percentage of correct prediction in the following manner:

(10) \begin{eqnarray*}TPGP= \frac{TTP+TFP}{TR} .\end{eqnarray*}

*TPGP*: Total percentage of good predictions

*TTP*: Total of true positives

*TFP*: Total of false positives

*TR*: Total of records

Validation of this modelling was performed using field occurrence data of the vector *M. persicae* and the virus PVY-O from the CABI database, only for Africa (coordinates are approximate and represent the central point of each location). This continent was selected because Africa is of vital importance for the study of Potato virus Y, not as a region with the highest potato production, but as a continent with the highest rate of food insecurity. PVY is transmitted non-persistently by many aphid species (such as *M. persicae*). African ecosystems host a wide range of aphid vectors, and their population dynamics are influenced by unique climatic conditions. Research in Africa helps scientists understand how PVY spreads in environments with high vector pressure and how climate change might influence future epidemics (Gildemacher et al., 2009).

Collecting actual absence data in the field (of both the vector and the virus in question) can present errors, such as accessibility to all potential areas, limited altitudinal variability, timing of assessment, phenological stage of the crop under inspection, and insufficient study efforts, among others. Then an alternative is to use background points, which do not represent explicit absence data but rather encompass habitats as unsuitable based on several measurable criteria (altitude, intrinsic rate range, remoteness from presence points, *etc*.) (Hijmans & Elith, 2021; Hijmans, 2001). These background data do not attempt to predict absence locations, but rather to characterize the environments in the study region (Phillips et al., 2009). To optimize the selection of background point values, we selected temperature values that are outside the limits of the intrinsic rate (*r*_*m*_), estimated by the ILCYM software; then locations are selected where the temperature does not allow the population of that species to grow under ideal environmental conditions (Gamarra et al., 2020).

### Virus transmission index

ILCYM can calculate the virus transmission index (VTI) of the insect vector. These indices help identify areas vulnerable to pest infestation that have the capacity to transmit a certain virus, thus allowing the development of a risk alert system through the interpretation of the critical values produced by the software, following the methodology described by Gamarra et al. (2020).

ILCYM calculates a specific transmission risk using the values obtained from immature survival, reproduction, transmission rate and their respective formulas as follows:

(11) \begin{eqnarray*}M(T)& =h(T)\end{eqnarray*}

(12) \begin{eqnarray*}IS(T)& =1-M(T)\end{eqnarray*}

(13) \begin{eqnarray*}TO(T)& =g(T)\end{eqnarray*}

(14) \begin{eqnarray*}TR(T)& =d(T)\end{eqnarray*}

(15) \begin{eqnarray*}VTIs(T)& =f(IS(T),TO(T),TR(T))\end{eqnarray*}

where *T* is the air temperature, *M* is the mortality (Total mortality of all immature stages), *IS* is the immature survival, *TO* is the total oviposition, *TR* is the transmission rate.

Hence:

(16) \begin{eqnarray*}VTI= \frac{\sum _{i=1}^{n}VTIs \left( {T}_{i} \right) }{n} \end{eqnarray*}

n = number of temperature records for 1 full year.

To develop global level transmission risk maps, ILCYM simultaneously extracted the monthly maximum and minimum temperature data for one year (12 sets of monthly data starting from January to December) with their respective geographical coordinates from the WorldClim 2.1 database (https://worldclim.org/data/worldclim21.html).

(17) \begin{eqnarray*}{T}_{min}\{ lon,lat\} _{i};i=1,2,\ldots \ldots ,12.\end{eqnarray*}

This represents the minimum temperature where, “*lon*” is the longitude and “*lat*” is the latitude respectively.

Temperature data were arranged into 24 matrices based on longitude and latitude, split into 12 for minimum and 12 for maximum temperatures. Each geographical point was then represented in a table with two columns, holding minimum and maximum temperatures for each month. These tables facilitated spatial phenological simulations. Correspondingly, for every risk index calculated, an additional matrix was generated with identical longitude and latitude organization. These matrices were then converted into ASCII files and imported into the R programming environment, which was equipped with GIS capabilities, for the purpose of mapping and visualize the VTI index.

(18) \begin{eqnarray*}VTI \left\{ lon,lat \right\} =f \left( VTIs \left( Tmi{n}_{1}^{{}^{{^{\prime}}}} \right) ,.,VTIs \left( Tmi{n}_{12}^{{}^{{^{\prime}}}} \right) ,VTIs \left( Tma{x}_{1}^{{}^{{^{\prime}}}} \right) ,..,VTIs \left( Tma{x}_{12}^{{}^{{^{\prime}}}} \right) \right) \nonumber\\\displaystyle \end{eqnarray*}

where, $Tmi{n}_{i}^{{}^{{^{\prime}}}}=Tmin{ \left\{ lon,lat \right\} }_{i}$ and $Tma{x}_{i}^{{}^{{^{\prime}}}}=Tmax{ \left\{ lon,lat \right\} }_{i}$, with *i* = 1, 2, .., 12

The distribution of *M. persicae* in potato-growing areas was assessed using global potato production regions. These regions were established by the International Potato Center (Hijmans, 2001). Areas were mapped using a 10-minute grid resolution to identify the regions at risk of virus transmission based on the potential establishment of *M. persicae* populations in areas where potatoes are present.

## Results

### *Myzus persicae* nymphal longevity, survival, and development rate

A comprehensive analysis of the development time, longevity, and survival of the nymphal stage of the aphid *M. persicae* at temperatures ranging from 10 °C to 30 °C was conducted using GLM models (Table 1) and the ILCYM software (Table S1). For nymph longevity, the longest duration was observed at 10 °C, averaging 12.58 days, which decreased steadily with increasing temperatures, reaching the lowest at 30 °C with an average of 9.74 days. Nymph survival was lowest at extreme temperatures, with 58% survival at 10 °C and 46% survival at 30 °C (Table S1).

**Figure 1:**
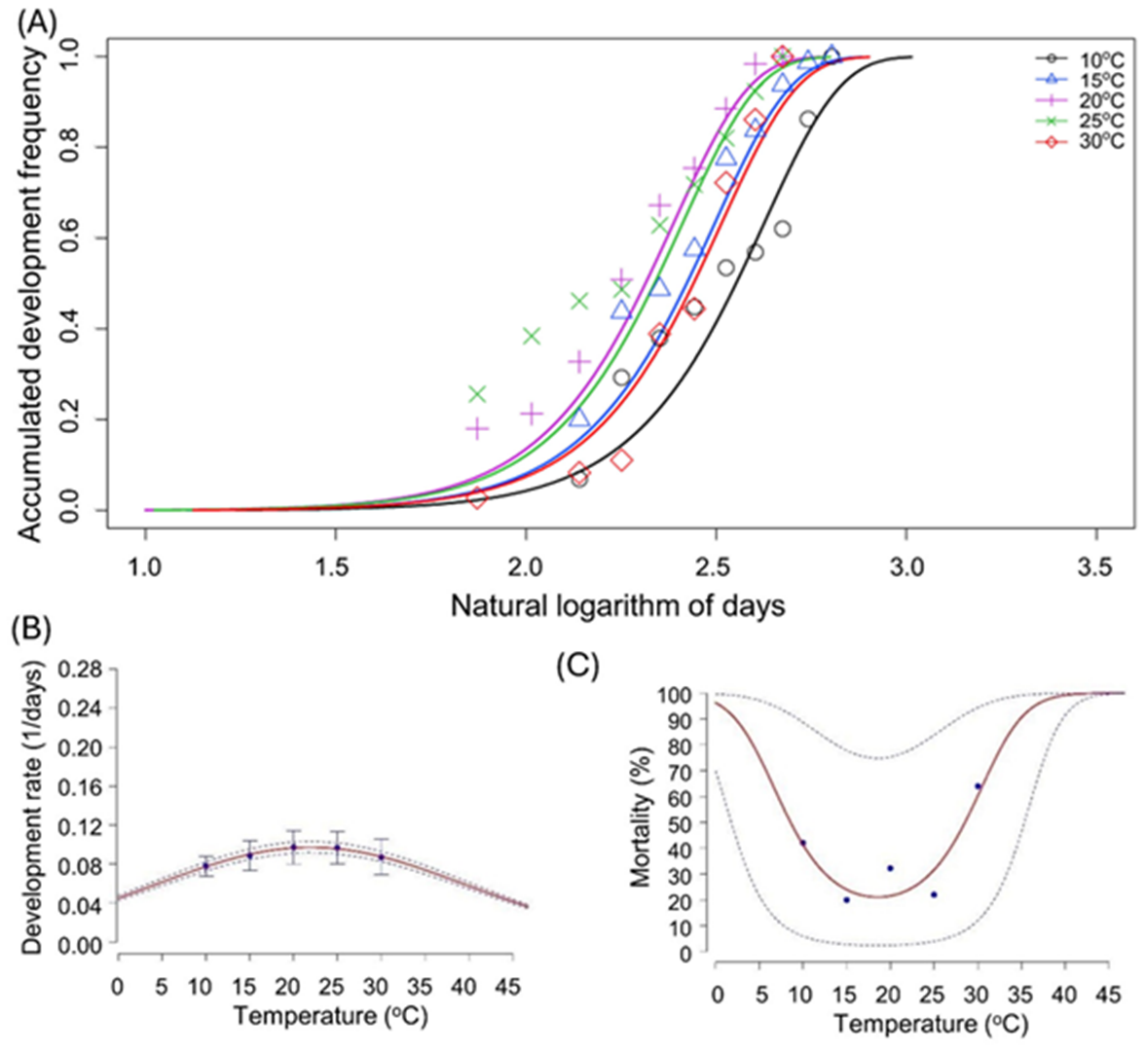
Temperature-dependent cumulative distribution of development time, development rate and mortality rate of nymph life stage of *Myzus persicae.* (A) Cumulative distribution frequency of nymphal development time fitted to Weibull models across five constant temperatures (10 °C, 15 °C, 20 °C, 25 °C, and 30 °C). Symbols represent observed data, and solid lines represent model fits. (B) Temperature-dependent development rate (1/days), showing mean values with standard error bars across the five tested temperatures. The curve was fitted using the Taylor model. (C) Mortality rate of nymphs at different constant temperatures, fitted with a quadratic function. Symbols represent observed data dashed lines indicate 95% confidence intervals around the fitted curve.

The variation in development times during the immature life stage across all temperatures was best described using the Weibull function (Table S1). This approach yielded highly significant common scale parameters (*p* < 0.001), as indicated by the lower AIC value, effectively capturing the variability in nymph development with respect to temperature. The common slopes were also highly significant (*p* < 0.001), providing an adequate description of the overall variability in the development of immature life stages (Fig. 1A). A linear model failed to describe the development rate appropriately at extreme temperatures, so a nonlinear model was fitted using AIC selection criteria. The temperature-dependent median developmental rate was accurately modelled by the Taylor model (Table S2). This model explained more than 97% of the variation in median development times due to temperature. The parameter *Topt* in the Taylor model indicated that the fastest development rate occurred at 23 °C (Table S2, Fig. 1B). Significant differences in mortality were observed across different temperatures (*F* = 3.04, *df* = 2, *p* = 0.247). Mortality rates were predicted to be highest at extreme temperatures, with 94% mortality at 0 °C and 100% mortality at 45 °C, while the lowest mortality was recorded at 20 °C. The effects of temperature on the mortality of *M. persicae* in the immature stage was best described by a quadratic model (Table S3 ; Fig. 1C). This model predicted increased mortality as temperatures deviated from the optimal range, indicating survival limits around 5 °C and 35 °C (Fig. 1C).

**Table 1:** Nymphal and adult developmental time and fecundity of *M. persicae* across five constant temperatures.

Temperature (°C)	Nymph development time	Adult longevity	Total life time	Fecundity (offspring per female)
10 °C	12.58 ± 0.38 ** a**	12.46 ± 0.53 ** a**	25.04 ± 0.56 ** a**	29.81 ± 1.83 ** a**
15 °C	11.50 ± 0.36 ** b**	13.48 ± 0.53 ** a**	24.48 ± 0.49 ** b**	45.82 ± 2.32 ** b**
20 °C	11.00 ± 0.26 ** c**	11.05 ± 0.50 ** b**	21.37 ± 0.56 ** c**	39.68 ± 2.08 ** c**
25 °C	10.32 ± 0.32 ** d**	9.21 ± 0.42 ** c**	18.96 ± 0.46 ** d**	38.59 ± 2.51 ** c**
30 °C	9.74 ± 0.33 ** e**	6.44 ± 0.40 ** d**	17.94 ± 0.54 ** e**	14.25 ± 1.09 ** d**
LR*χ*^2^	26.11	111.98	104.81	124.29
*df*	4	4	4	4
*p*	<0.0001	*p* < 0.0001	*p* < 0.0001	*p* < 0.0001

**Figure 2:**
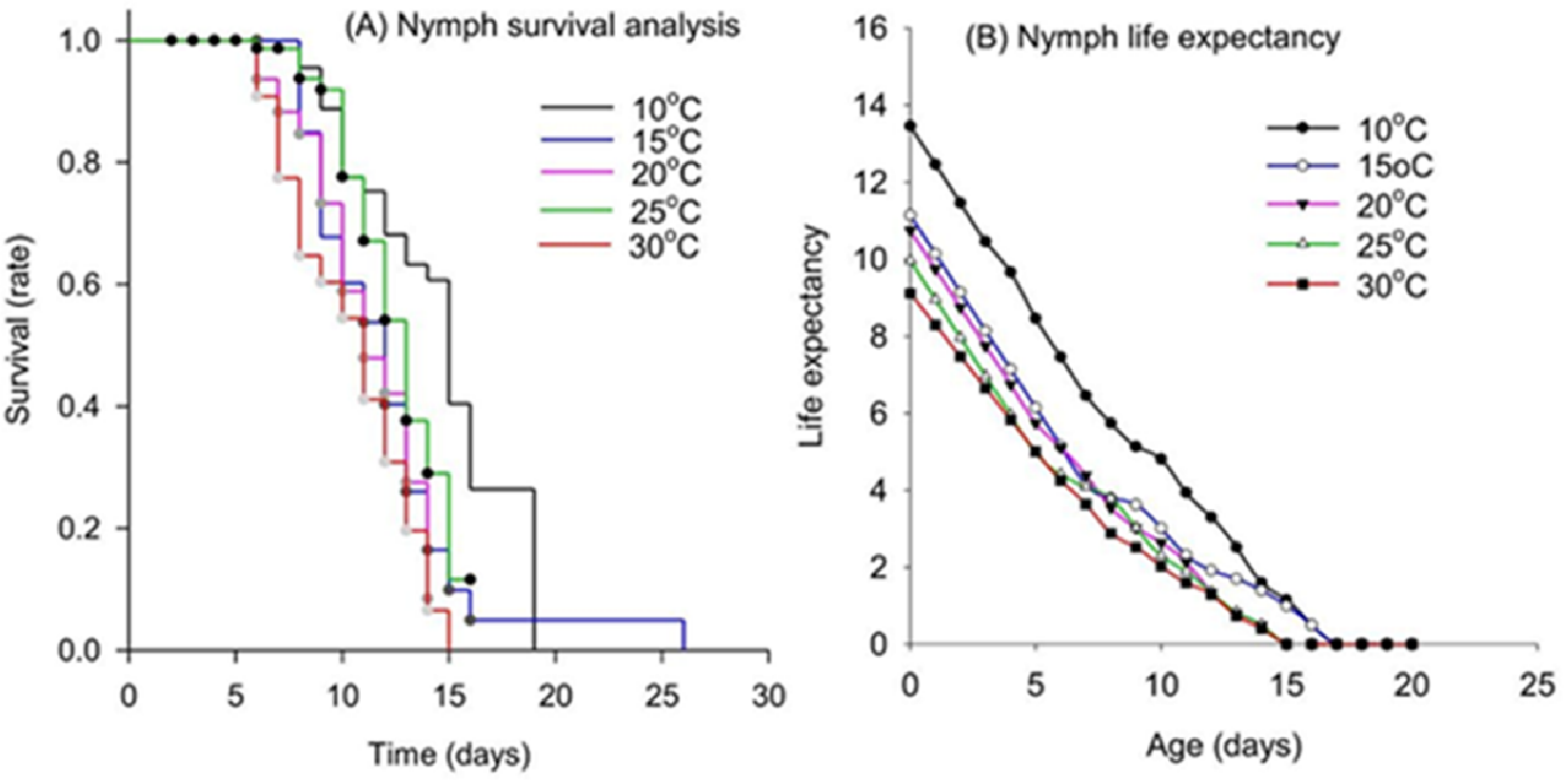
Temperature-dependent survival rate and life expectancy of nymph life stage of *Myzus persicae*. (A) Kaplan–Meier survival analysis of nymphs reared at five constant temperatures (10 °C, 15 °C, 20 °C, 25 °C, and 30 °C). (B) Age-specific life expectancy of nymphs reared at five constant temperatures (10 °C, 15 °C, 20 °C, 25 °C, and 30 °C).

Furthermore, temperature significantly influences survival trends (Fig. 2A). At 10 °C, nymphs exhibit the highest resilience, maintaining nearly 100% survival until around day 9, after which their survival gradually declines to 0% by day 19 (Fig. 2A). As the temperature increases, the survival rate declines more rapidly. At 15 °C, the survival rate drops below 10% by day 15 and reaches 0% by day 26 (Fig. 2A). This decline becomes sharper at 20 °C, 25 °C, and 30 °C, with survival falling to 0% by day 14, 16, and 15, respectively, highlighting the critical temperature sensitivity of the nymph stage to increased thermal conditions (Fig. 2A). Figure 2B presents life expectancy trends of *M. persicae* nymphs across temperatures ranging from 10 °C to 30 °C. Life expectancy decreases with age, but the rate of decline is influenced by temperature. At 10 °C, life expectancy starts the highest, suggesting slower developmental rates or better survival at lower temperatures. As temperature increases, life expectancy starts lower and decreases more rapidly. At 30 °C, nymphs show the lowest initial life expectancy, which rapidly declines, reaching near zero by about day 15 (Fig. 2B).

### *Myzus persicae* adult longevity, survival, and life expectancy

For adult longevity, the longest duration is at 15 °C (13.48 days), which is not significantly different from 10 °C, and the shortest at 30 °C (6.44 days). Total longevity also decreases with temperature, from 25.04 days at 10 °C to 17.94 days at 30 °C (Table 1). Similar results and trends were obtained when data was analysed using ILCYM (Table S1).

**Figure 3:**
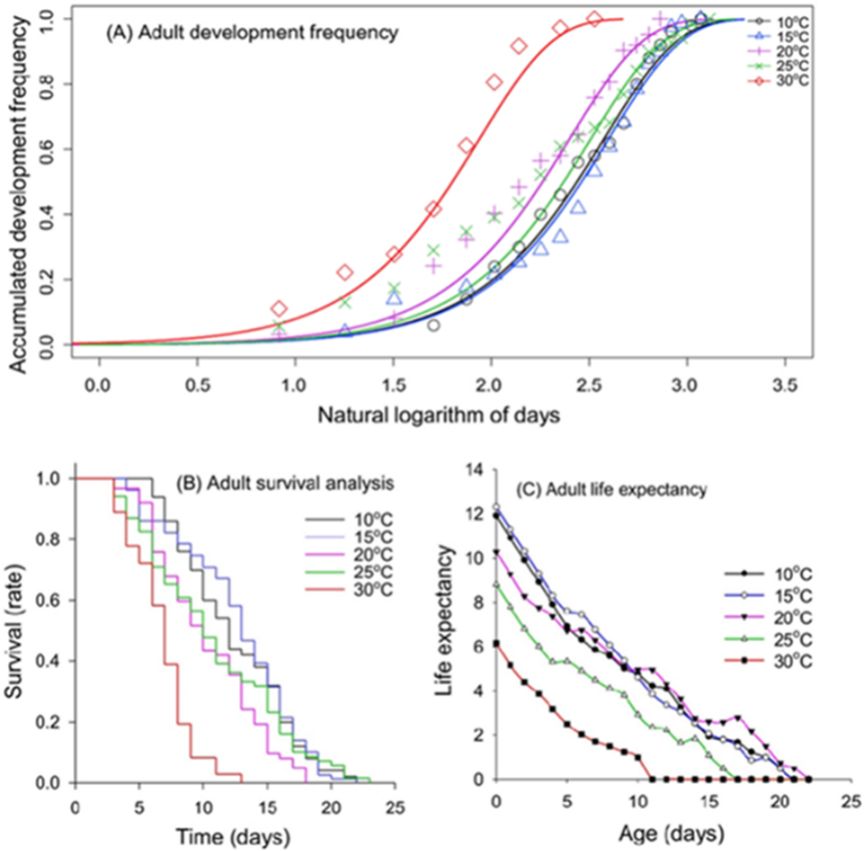
Temperature-dependent cumulative distribution of development time, survival rate and life expectancy of adult life stage of *Myzus persicae*. (A) Cumulative distribution of adult development time across constant temperatures (10–30 °C), fitted with Weibull functions. (B) Kaplan–Meier survival curves of adults across constant temperatures (10–30 °C). (C) Age-specific life expectancy of adults across constant temperatures (10–30 °C).

Figure 3A indicates that the development time increased with temperature up to a certain point, beyond which it declined, where the temperature becomes detrimental to development; this is optimally described using a Weibull distribution function (Table S1). This approach produced highly significant common scale parameters (*p* < 0.001), as indicated by the lowest AIC values, effectively capturing the variability in adult development in relation to temperature. Additionally, the common slopes were highly significant (*p* < 0.001), offering a robust description of the overall variability in the development of the adult life stages. The survival rate was distinctly influenced by temperature, demonstrating clear temperature-dependent survival patterns (Fig. 3B). At a lower temperature of 10 °C, adults exhibit a gradual decline in survival, maintaining over 50% survival up to about day 12, and then decreasing to approximately 25% survival by day 16, and reaching 0% at day 22 (Fig. 3B). As temperatures increase, a marked acceleration in the rate of decline in survival is observed. At 20 °C, survival falls to about 50% at day 11 and reached 0% at day 18. Similarly, at 25 °C, survival falls to about 50% at day 11 and falls below 20% at day 16 (Fig. 3B). The trend becomes more pronounced at 30 °C, where survival steepens further, dropping below 40% at day 7 and with survival plummeting to 0% by day 13 (Fig. 3B). Similarly to immature life stage, life expectancy at adult stage at cooler temperatures (10 °C and 15 °C) starts higher and declines at a slower rate compared to warmer temperatures (Fig. 3C). The life expectancy at 25 °C and especially 30 °C shows a steeper decline. At 30 °C, adults have a significantly shorter life expectancy, with values plummeting to near zero by day 11 (Fig. 3C).

### Adult senescence rate and fecundity of *Myzus persicae*

**Figure 4:**
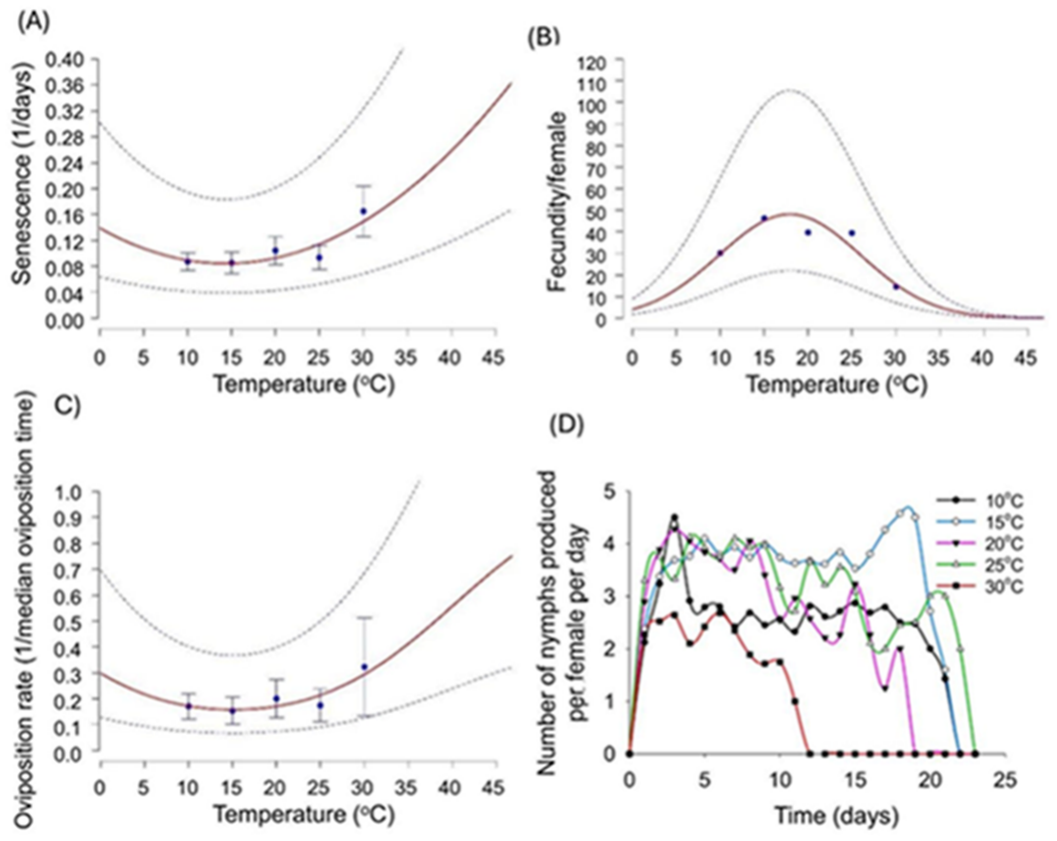
Temperature-dependent senescence rate, total fecundity per female, oviposition rate and daily fecundity of *Myzus persicae*. (A) Temperature-dependent senescence rate of adult females. (B) Total fecundity (nymphs per female per life cycle). (C) Relationship between temperature and survival time/oviposition rate, best described by exponential models. (D) Daily reproductive output per female. All variables were assessed across five constant temperatures (10 °C, 15 °C, 20 °C, 25 °C, and 30 °C).

A quadratic model was employed to explore the relationship between the senescence rates of adults and temperature (Fig. 4A and Table S5). The lowest senescence rates were recorded within the temperature range of 10–20 °C (Fig. 4A). Female fecundity showed a significant decrease with increasing temperature either using a negative binomial GLM (Table 1) or the ILCYM software (Table S4). At 10 °C, the average fecundity is 29.81 and 30.16 offspring per female respectively, which is the highest recorded, while at 30 °C, it drastically drops to only 14.25 and 15.66 offspring per female respectively (Table 1 and Table S4). This pattern suggests that lower temperatures favour higher reproductive output. The Taylor function demonstrated a significant impact of temperature on oviposition time. The effects of temperature on fecundity were best captured by a quadratic model, which predicted the highest fecundity at 15−20 °C (Fig. 4B and Table S5). Additionally, the relationship between temperature and both the survival time of adults and the oviposition rate were most accurately described by an exponential model (Fig. 4C and Table S5).

Figure 4D depicts the reproductive output, measured as the number of nymphs produced per female per day, of *M. persicae* across different temperature conditions. Each temperature condition, represented by distinct lines (10 °C, 15 °C, 20 °C, 25 °C, and 30 °C), illustrates unique trends in nymph production. At the lowest temperature of 10 °C, nymph production starts at about 1 nymph per female per day, fluctuates, and peaks around day 3 before gradually declining. This temperature maintains relatively consistent, albeit declining, reproductive output until it drops sharply after day 20. The reproductive output at 15 °C shows initial fluctuations with several peaks and valleys but generally trends higher compared to 10 °C, it peaks (average five nymphs/female/day) around day 19, after which it drops significantly. At 20 °C, the pattern starts similarly with fluctuations, reaching several peaks which are higher than those observed at cooler temperatures. The reproductive output remains relatively high and stable until it begins a steep decline around day 18. In the 25 °C condition, nymph production starts higher than in all cooler conditions, peaks early, and maintains a high output longer, until a sharp decline after day 15, suggesting that while higher temperatures may initially promote greater reproductive output, they also lead to quicker declines. At the highest temperature of 30 °C, nymph production is initially low, peaks slightly around day 7, and falls to zero by day 12, indicating that extremely high temperatures may severely inhibit reproductive capacity.

### Life table parameters of *Myzus persicae*

Table 2 provides detailed life table parameters of the aphid *M. persicae* across a range of temperatures from 10 °C to 30 °C, illustrating the effects of temperature on reproductive and survival metrics. At 10 °C, the net reproduction rate (R_o_) is relatively low at 170.36, and it increases significantly at 15 °C to 365.80, indicating more favourable conditions for reproduction at this slightly warmer temperature. However, as the temperature increases further to 20 °C and 25 °C, R_o_ showed varied responses, with a decrease at 20 °C (219.48) and a moderate increase at 25 °C (272.30), followed by a drastic drop at 30 °C (51.30). The intrinsic rate of increase (r_m_) also varied with temperature, starting at 0.76 at 10 °C and peaking at 0.98 at 30 °C. The finite rate of increase (*λ*) follows a similar trend, gradually increasing with temperature. Mean generation time (T) decreased as temperature increases, from 6.84 days at 10 °C to 3.96 days at 30 °C, showing that higher temperatures accelerate life cycle completion. Doubling time (DT), which indicates the time it takes for the population to double, also decreases with rising temperature, from 0.93 days at 10 °C to 0.71 days at 30 °C.

**Table 2:** Mean ± SE of life table parameters of *M. persicae* across five constant temperatures.

Temperature (°C)	Net reproduction rate (*R*_*o*_)	Intrinsic rate of increase (*r*_*m*_)	Finite rate of increase (*λ*)	Mean generation time (*T*)	Doubling time (*DT*)
10 °C	170.36 ± 16.4 ** a**	0.76 ± 0.03 ** a**	2.14 ± 0.07 ** a**	6.84 ± 0.3 ** ab**	0.93 ± 0.04 ** a**
15 °C	365.80 ± 37.1 ** b**	0.77 ± 0.02 ** a**	2.16 ± 0.05 ** a**	7.69 ± 0.3 ** a**	0.91 ± 0.03 ** a**
20 °C	219.48 ± 35.9 ** ac**	0.96 ± 0.04 ** ab**	2.62 ± 0.12 ** b**	5.66 ± 0.3 ** c**	0.74 ± 0.03 ** b**
25 °C	272.30 ± 37.8 ** abc**	0.85 ± 0.03 ** bc**	2.36 ± 0.07 ** ab**	6.56 ± 0.3 ** bc**	0.82 ± 0.03 ** ab**
30 °C	51.30 ± 4.7 ** c**	0.98 ± 0.05 ** c**	2.78 ± 0.15 ** b**	3.96 ± 0.2 ** d**	0.71 ± 0.04 ** b**
	*χ*^2^= 32.47	*F* = 8.44	*χ*^2^ = 21.26	*F* = 24.24	*F* = 8.63
	*df=* 4	*df=* 4	*df=* 4	*df=* 4	*df=* 4
	*p* < 0.0001	*p* < 0.0001	*p* < 0.0001	*p* < 0.0001	*p* < 0.0001

### PVY acquisition and transmission by *M. persicae* to potato plants

**Figure 5:**
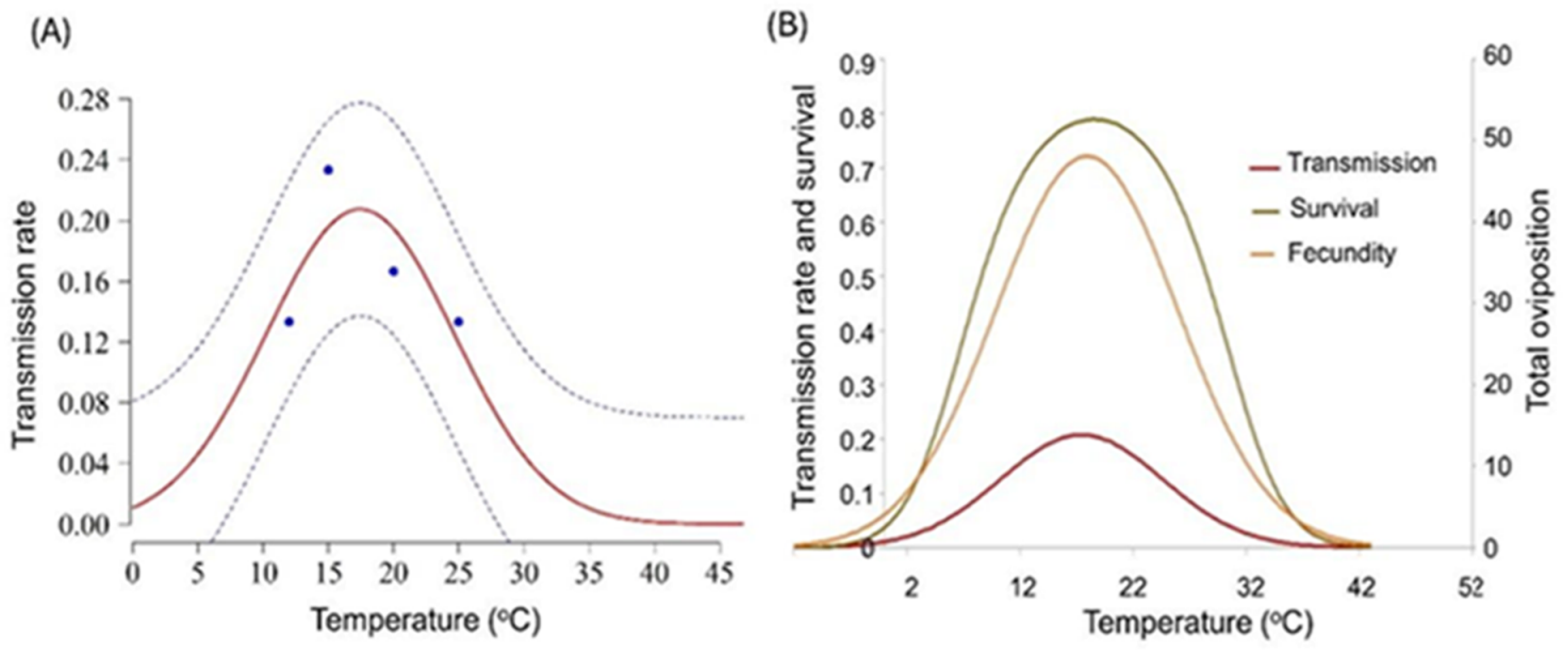
Transmission rates of potato virus Y (PVY) by *Myzus persicae.* (A) Transmission rates of PVY across temperatures, fitted with the Taylor model. (B) Interaction between transmission efficiency, survival, and fecundity across temperatures.

The temperature-dependent transmission rate of PVY by the aphid *M. persicae* was best captured by the Taylor model, which predicted the highest transmission rate between 15 °C and 20 °C (Fig. 5A and Tables S6 and S7). This optimal range of temperatures yields the highest transmission rate. At temperatures below and above this range, the transmission rate declines, with the lowest rates observed near 0 °C and 45 °C. Figure 5B presents the interaction between the transmission rate of PVY, survival, and total oviposition of *M. persicae* across different temperatures. The transmission rate of PVY increases with temperature, peaking around 18 °C, and declines at higher temperatures, indicating optimal transmission efficiency at moderate temperatures. The survival rate of *M. persicae* reaches its maximum between 20 °C and 25 °C, decreasing significantly at both lower and higher temperatures. Similarly, fecundity, or total oviposition, peaks between 18 °C and 22 °C and diminishes outside this optimal range, showing that reproductive output is highest at moderate temperatures. Notably, the optimal temperature for the highest PVY transmission rate slightly precedes the peak survival and fecundity rates of *M. persicae*. This suggests that while moderate temperatures favour virus transmission, slightly warmer temperatures are more conducive to aphid survival and reproduction.

### Establishment Risk Index, Generation Index, Virus Transmission Index, and potential PVY transmission across the globe

**Figure 6:**
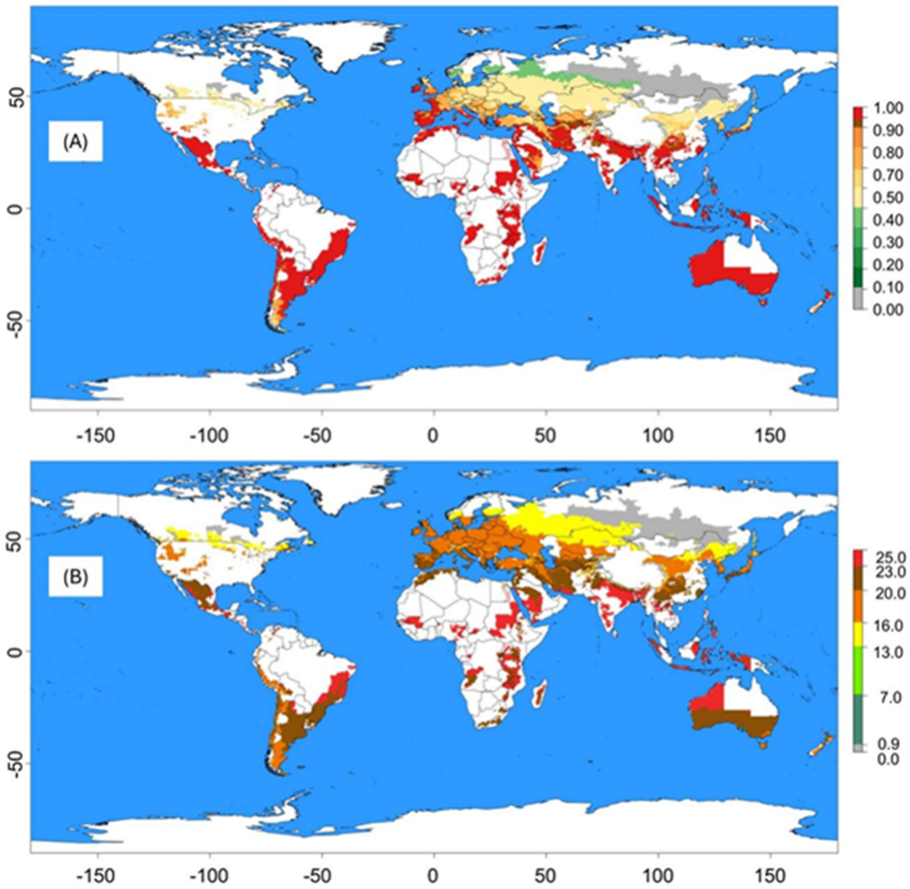
Global establishment risk and generation indices of *Myzus persicae*. (A) Establishment Risk Index (ERI), derived from temperature-dependent phenology model. (B) Generation Index (GI). Together, these indices demonstrate the global potential for *M. persicae* persistence and PVY transmission under current climatic conditions.

Figure 6 illustrates the Establishment Risk Index (ERI) (A) and Generation Risk Index (GRI) (B) of *M. persicae* in potato growing regions based on global climate temperature data. Regions with the highest ERI (0.90–1.00) indicate a very high likelihood of *M. persicae* establishment. These areas include tropical and subtropical regions across South America, Africa, South Asia, Southeast Asia, and parts of Oceania. Moderate-risk regions (0.40–0.80) are found in temperate zones, including parts of North America, Europe, and East Asia. Low-risk areas (0.10–0.30) are primarily located in higher latitudes and mountainous regions with cooler temperatures. Very low-risk zones (0.00–0.10) are found in extremely cold climates such as the Arctic, Antarctic, and high-altitude regions (Fig. 6A).

Regions with the highest Generation Index (GI) (23.0–25.0 generations) indicate a high potential for multiple generations per year. These areas are predominantly in tropical and subtropical regions of South America, Africa, South Asia, Southeast Asia, and parts of Oceania. Moderate GI regions (13.0–20.0 generations) are mainly in temperate zones, including parts of North America, Europe, and East Asia. Low GI areas (7.0–13.0 generations) are in higher latitudes and mountainous regions with cooler temperatures. Very low GI zones (0.0–7.0 generations) are found in extremely cold climates like the Arctic, Antarctic, and high-altitude regions (Fig. 6B).

**Figure 7:**
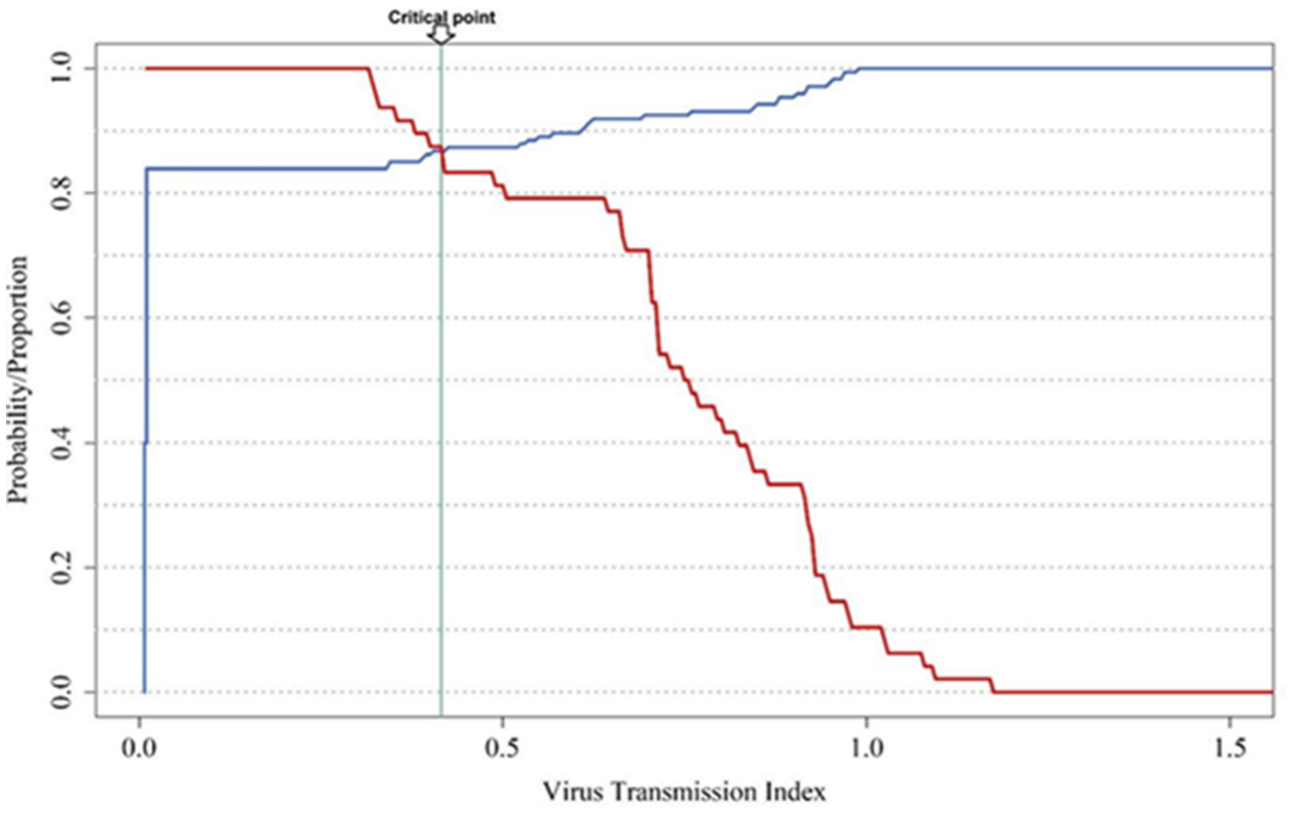
Sensitivity (true positive rate) *versus* specificity (true negative rate) for field survey data for the Virus Transmission Index (VTI).

**Figure 8:**
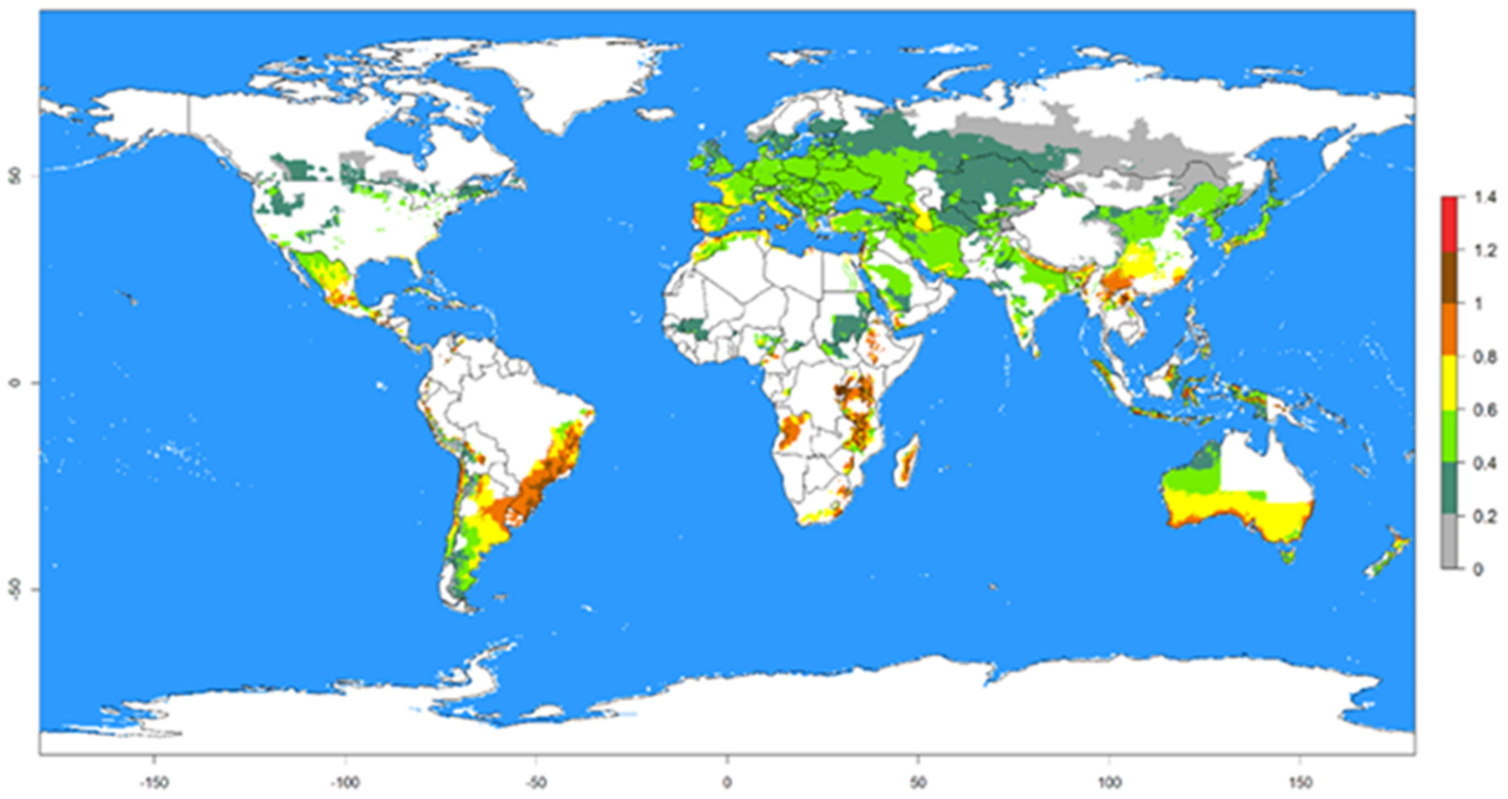
Global Virus Transmission Index (VTI) for PVY-O spread by *Myzus persicae* in 2021. Global VTI for PVY-O spread by M. persicae in 2021, based on climate temperature data from WorldClim. WorldClim provides high-resolution global climate layers, including monthly temperature, widely used for ecological and agricultural modeling.

We assess sensitivity, also known as the true positive rate, and specificity, known as the true negative rate, to describe the accuracy of the values used in the Virus Transmission Index (VTI) (Fig. 7). Figure 8 illustrates the VTI of PVY by *M. persicae* based on global climate temperature data. Regions with the highest VTI values (1.2–1.4) indicate a very high potential for virus transmission, primarily located in tropical and subtropical regions of South America, Central Africa, South Asia, and Southeast Asia. Areas with moderate VTI values (0.6–1.2) are found in parts of North America, South America, Africa, Europe, and East Asia, exhibiting a significant potential for virus transmission. Regions with low VTI values (0.2–0.6) are mainly situated in temperate zones, including parts of North America, Europe, and East Asia, while very low VTI areas (0.0–0.2) are typically located in extremely cold climates, such as the Arctic, Antarctic, and high-altitude regions (Fig. 8).

**Figure 9:**
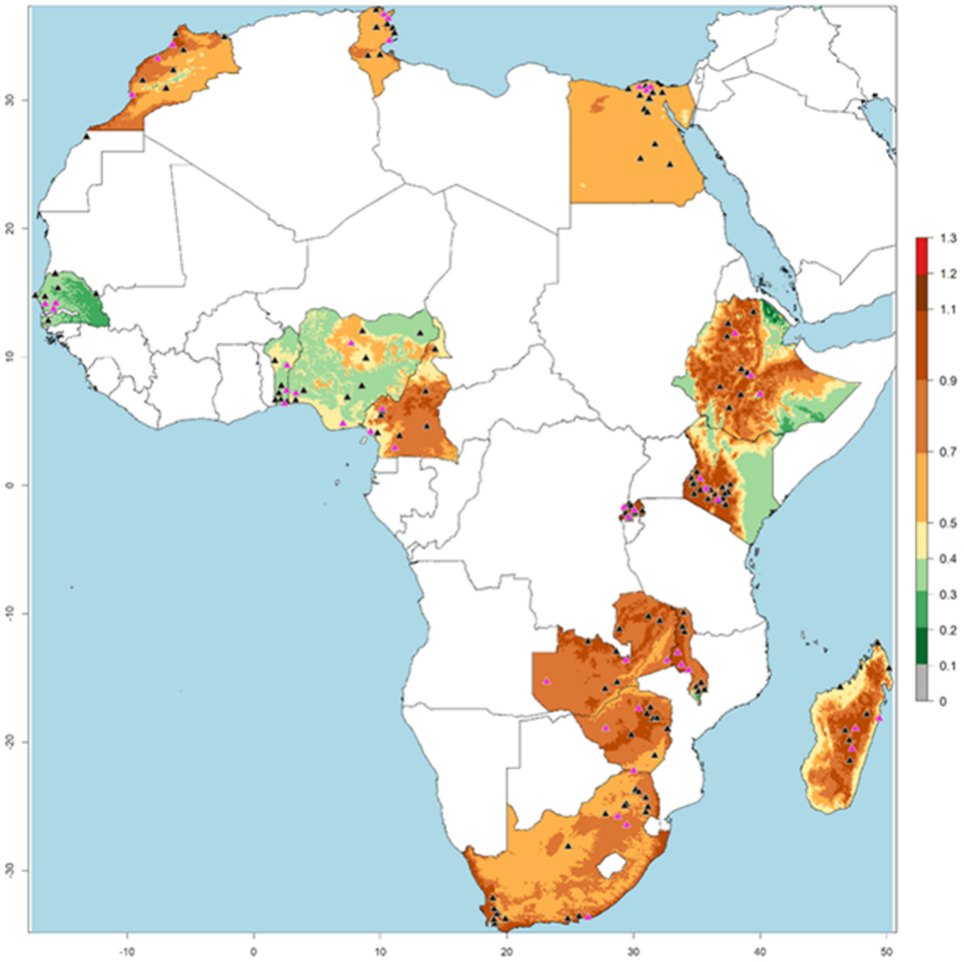
Virus Transmission Index (VTI) of PVY-O transmitted by *Myzus persicae* in Africa (2021). Virus Transmission Index (VTI) for PVY-O spread by *M. persicae* in Africa (2021), based on climate temperature data from WorldClim. Pink triangles indicate PVY-O presence, and black triangles indicate *M. persicae* presence, as recorded in the CABI database.

African risk map (Fig. 9) generated from VTI using current climate data identify regions at significant risk for PVY-O epidemics, particularly where VTI values exceed 0.415 indicating temperatures conducive to both *M. persicae* population growth and high PVY transmission efficiency. This map confirms established PVY presence in specific strains like PVY-O documented in Africa. It is also observed that the vast majority of these points are located within areas with a VTI value above 0.415 (shown in colors from yellow to brown), indicating a strong spatial correspondence between the known presence and the model’s prediction (VTI >  0.415). A higher degree of this correspondence is notable in Morocco, Tunisia, Cameroon, Egypt, Kenya, Ethiopia, Malawi, Zambia, Zimbabwe, Madagascar, and South Africa.

By evaluating all PVY-O occurrence locations in Africa, it was determined that the favorable temperature range for the reproductive and survival potential of *M. persicae* lies between approximately 5 °C and 32 °C, and also includes areas below 1,828 m above sea level (90th quartile).

## Discussion

Although many unknowns remain about specific effects of global warming, it is widely accepted that it significantly affects crops, such as potatoes, as well as the insect pests associated with them, like aphids. This is likely through the effects of climatic variables, especially temperature. Temperature is a critical factor influencing the lifespan, survival, and development of the green peach aphid, *M. persicae*. Our study demonstrates that the developmental time of *M. persicae*, including nymph and adult longevity, is significantly affected by temperature. The longest life span was observed at the lowest temperatures (10 °C and 15 °C), while the shortest lifespan occurred at the highest temperature (30 °C). The study by Baral et al. (2022), which assessed the effect of temperatures ranging from 17 °C to 29 °C on *M. persicae*, also found that longevity decreases as temperature increases. However, their study reported longer lifespans for *M. persicae* compared to ours, which may be attributed to their use of cabbage (*Brassicae olerasia*) as a host plant (Baral et al., 2022) or different origin of the aphid colony. Additionally, the study of (Khurshid et al., 2022) showed that higher temperatures are detrimental to the survival and reproduction of *M. persicae*.

The survival and life expectancy trends across different temperatures reveal that cooler temperatures favour higher survival rates and longer life expectancy for both nymph and adult stages. At 10 °C, nymphs exhibited nearly 100% survival up to around day 9, followed by a gradual decline until reaching 0% by day 19. In contrast, survival at 30 °C plummeted to 0% by day 15 (Fig. 2A). This indicates that extreme temperatures, particularly high temperatures, have a detrimental impact on aphid survival. Multiple studies have concluded that temperature extremes affect insect development, fecundity, survival, and geographic distribution (Régnière, St-Amant & Duval, 2012; Ju, Wang & Li, 2011; Abarca & Spahn, 2021). At 10 °C, adult aphids maintain over 50% survival up to about day 12, with a gradual decline to approximately 20% by day 17. As temperatures increase, the survival rate declines more rapidly, dropping to below 10% by day 9 at 30 °C (Fig. 3B). These findings suggest that while moderate temperatures favour aphid longevity, higher temperatures significantly reduce their life expectancy. This underscores the need for temperature-specific management strategies in pest control (Scherm, 2004).

Fecundity of *M. persicae* demonstrates a classic hump-shaped relationship with temperature. At 10 °C, the average fecundity is 29.81 nymphs per female, with a peak at 15 °C (45.82 nymphs/female). After this point, fecundity decreases, with the lowest value (14.25 nymphs per female) at 30 °C. This pattern suggests that intermediate temperatures favour higher reproductive output. The relationship between temperature and fecundity was best described by the Wang 11 model, which predicted the highest fecundity at 15–20 °C. Life table parameters of *M. persicae*, including net reproduction rate, intrinsic rate of increase, and mean generation time, are significantly influenced by temperature. The net reproduction rate is highest at 15 °C, indicating favourable conditions for reproduction at this temperature. However, as temperature increases beyond this point, net reproduction rate decreases, with the lowest value observed at 30 °C. The intrinsic rate of increase peaks at 30 °C, suggesting that the population growth rate is highest at the warmest temperature despite the lower net reproduction rate. These findings highlight the complex interactions between temperature and aphid population dynamics, underscoring the need for predictive models to inform pest management practices under varying climatic conditions (Intergovernmental Panel on Climate Change (IPCC), 2015; Abarca & Spahn, 2021).

These observations are consistent with previous studies that have documented the impact of temperature on aphid reproduction. For instance, the study by Ahn et al. (2020) on *Acyrthosiphon pisum* (pea aphid) showed highest fecundity at 15 °C and 20 °C (74.9 and 62.5 nymphs/female, respectively) and significantly decline as temperature increases with the lowest at 30 °C (4.5 nymphs/female) using *Vicia faba* as a host plant. The study of Cividanes & Souza (2003) in *M. persicae* showed highest fecundity at 15 °C (69.2 nymphs/female) and lowest fecundity at 25 °C (30.7 nymphs/female) using *B. oleracea* as a host plant. However, the study of Baral et al. (2022) showed higher fecundity of *M. persicae* at 23 °C (72.4 nymphs/female); fecundity decreased below and upper this threshold with the lowest at 29 °C (28 nymphs/female) in *B. oleracea*. These findings are further supported by the work of Leather et al. (1989), who reported that temperature and host species play a critical role in the reproductive success of aphids. Their research found that the aphid *Rhopalosiphum padi* fecundity is higher at 15 °C when the host is either oats cv. Aster (38.2 nymphs/female) or timothy grass (33.6 nymphs/female) and fecundity decreases when temperature is 20 °C when the host is either oats cv. Aster (28.47 nymphs/female) or wheat cv. Huntsman (25.14 nymphs/female) (Leather, Walters & Dixon, 1989). Moreover, other studies have explored the physiological mechanisms underlying this temperature-dependent fecundity pattern. Dixon (1977) suggested that lower temperatures might prolong the developmental period of aphids, allowing them more time to produce offspring. Conversely, higher temperatures may accelerate development but at the cost of reduced fecundity and longevity. Although the intrinsic rate of increase (*r*_m_) peaked at 30 °C, this was due to the very short generation time at this temperature, which inflates rate-based growth estimates. In contrast, the net reproductive rate (*R*_0_) was lowest at 30 °C, reflecting reduced fecundity and survival. Together, these results indicate that while individual turnover may be rapid under heat stress, overall population sustainability is compromised, highlighting the need to interpret *r*_m_ and *R*_0_ jointly.

Our study reveals that the efficiency of virus transmission is closely linked to the aphids’ physiological state, which is temperature dependent (Fig. 5B). Ng & Perry (2004) demonstrated that moderate temperatures enhance aphid feeding activity and mobility, thereby increasing the likelihood of virus transmission. We found that PVY transmission by *M. persicae* was highest at 20 °C, supporting the optimal physiology and behaviour for virus transmission. In comparison, Nancarrow et al. (2014) showed that the transmission of Barley yellow dwarf virus (BYDV), which is persistently transmitted by *Rhopalosiphum padi*, was highest at temperatures 10–21.1 °C (night-day fluctuations) compared to 5–16 °C (night-day fluctuation) and additionally that the potyvirus PVA is better transmitted when acquisition occurs at about 20 °C (van Munster, 2020). The temperature-dependent pattern of PVY transmission by *M. persicae* can likely be attributed to physiological and behavioural mechanisms. At optimal temperatures, aphids exhibit increased feeding frequency and duration, enhancing virus acquisition and transmission. Van Emden & Harrington (2017) explained that temperature affects the metabolic rate of aphids, influencing their feeding behaviour and transmission efficiency. Additionally, Van Munster (2020) noted that the stability and infectivity of plant viruses within aphid vectors can be temperature-dependent, with moderate temperatures maintaining virus viability and extreme temperatures degrading viral particles, reducing transmission efficiency. Besides that, PVY is transmitted by aphids through direct, reversible binding of virus particles to the aphid stylet during feeding and salivation, a process mediated by the virus-encoded HC-Pro protein, which bridges the viral coat protein and aphid stylet receptors (Blanc et al., 1998; Dombrovsky, Reingold & Antignus, 2014) and thus temperature may also affect in the process to influence transmission efficiency. Although in this study temperature dependent transmission efficiency closely resembled those of aphid life table parameters, in a study of potato yellow vein virus (PYVV) transmission by the greenhouse whitefly, transmission efficiency followed a dissimilar trend than the whiteflies life parameters (Gamarra et al., 2020), indicating this can vary among virus-vector system and may need to be studied individually for each case.

Using the risk prediction tool from the ILCYM 4.0 software, we mapped the potential activity of PVY transmission by *M. persicae* based on global climate temperature data. Regions with the highest potential activity are primarily located in tropical and subtropical regions, including South America, Central Africa, South Asia, and Southeast Asia. Tropical and subtropical regions, which exhibit higher average temperatures and humidity, provide ideal conditions for *M. persicae* activity and PVY transmission. Studies by Radcliffe & Ragsdale (2002); Ragsdale, Radcliffe & Di Fonzo (2001) confirm that warmer climates enhance aphid population growth and virus transmission rates. The presence of diverse and abundant host plants in these regions further supports the establishment and proliferation of both the aphid vector and the virus. Similarly, Gamarra et al. (2020) highlighted the increased risk of PVY transmission in tropical regions due to the favourable climate for both the whitefly vector and virus persistence. Moderate-risk regions include parts of North America, Europe, and East Asia. These temperate zones experience seasonal variations that can influence aphid population dynamics and virus transmission. In these regions, warmer months provide suitable conditions for *M. persicae* activity, while cooler periods may limit their population growth and transmission potential. Bhoi et al. (2022) indicated that in temperate climates, integrated pest management strategies are essential during peak aphid activity periods to mitigate the risk of PVY spread. Low-risk areas are situated in temperate zones and extremely cold climates such as the Arctic and Antarctic. These regions have limited aphid activity due to unfavourable temperatures for *M. persicae* survival and reproduction. Extreme cold climates inhibit aphid population growth and virus transmission (Leather, Walters & Dixon, 1989; Dixon, 1977). The short growing seasons and harsh environmental conditions in these regions restrict the establishment and spread of PVY.

The risk map generated in this study (Fig. 6) correspond well with potato production regions in Kenya and Eastern Africa, where PVY is commonly reported, providing preliminary ecological relevance (Onditi, Nyongesa & vander Vlugt, 2022; Nyakio et al., 2024). However, it is important to note that our results represent potential risk under constant temperatures and do not incorporate additional drivers such as fluctuating thermal regimes, PVY strain diversity, host resistance, or the role of multiple vector species. In the field, the majority of PVY transmission is mediated by alate aphids, whose dispersal and transient probing behaviour can substantially amplify spread across plants and fields. While our greenhouse-derived colony provides a standardized baseline for model parameterization, further validation using long-term field data and integration of alate movement into spatial models will strengthen predictive accuracy and applicability. Although leaf-disc assays are widely applied in demographic studies and produce results comparable to whole plants (Khurshid et al., 2022; Ahn et al., 2020), direct validation in this pathosystem remains necessary. Furthermore, host genotype and aphid clone can strongly affect virus transmission (Pinheiro et al., 2017; Jayasinghe et al., 2022). These limitations notwithstanding, our findings provide a critical first step in linking aphid biology, virus transmission, and climate risk to inform integrated pest and disease management in East African potato systems.

Our model achieved a maximum accuracy of 86.9% in predicting PVY-O presence and absence across African countries where the virus has been reported, using a VTI threshold of 0.415 (Table 3; Fig. 7). Despite the primary role of infected seed tubers in long-distance PVY-O spread—enabling introduction even without local vector transmission—the VTI-based risk map for *M. persicae*-PVY-O interaction showed strong agreement between predicted high-risk zones and known endemic regions (Fig. 9).

**Table 3:** Confusion matrix showing the best overall prediction accuracy for the Virus Transmission Index (VTI).

Accuracy of prediction using VTI > 0.415: ((42 + 151)/222) *100 = 86.9%.		
	Presence	Absence
Estimated presence	42	23
Estimated absence	6	151
	Total	222

Our approach has some important limitations, some of which could be addressed by extending the models in future studies. One key limitation is the assumption of constant temperatures; our model does not incorporate daily or seasonal temperature fluctuations, which are common in nature and particularly pronounced in temperate regions. Nevertheless, it is important to note that even under constant temperature regimes, nonlinear relationships among insect traits can pose substantial challenges for accurate measurement and modeling (Mordecai et al., 2019; Villena et al., 2022). Additionally, photoperiod plays a critical role in regulating the reproductive cycle of aphids. Specifically, shorter day lengths trigger a switch from asexual (parthenogenic) reproduction to sexual reproduction, a transition that facilitates the production of eggs capable of overwintering. In temperate regions, aphid populations are typically holocyclic (completing their life cycle with a sexual phase that produces overwintering eggs) while in tropical regions, populations are often anholocyclic (asexual reproduction throughout the year) (Blackman & Eastop, 2000; Margaritopoulos et al., 2002). These different overwintering strategies confer adaptive advantages by enhancing aphid survival and dispersal across diverse climatic conditions. This plasticity underscores the importance of incorporating regional reproductive dynamics and environmental heterogeneity in future modeling efforts to improve predictive accuracy and ecological relevance. We also acknowledge that life-history experiments were conducted on potato cv. Shangi using a Kenyan *M. persicae* colony, while transmission assays were performed on potato cv. Yungay using a Peruvian *M. persicae* colony from CIP. This approach was necessitated by logistical constraints related to the simultaneous availability of virus-free and PVY-infected plants. Host cultivar and aphid genotype can strongly influence virus transmission (Pinheiro et al., 2017; Jayasinghe et al., 2022). These differences represent a limitation of our study, and we caution readers that the integration of life-history and transmission datasets should be interpreted as complementary rather than directly equivalent.

The temperature-dependent fecundity pattern of *M. persicae* has significant implications for integrated pest management (IPM) strategies. Understanding the optimal and suboptimal temperature ranges for aphid reproduction aids in predicting population dynamics and devising effective control measures. During moderate temperature periods, aphid populations may surge, necessitating timely interventions like biological control agents or selective insecticides, while extreme temperatures may naturally limit reproduction, reducing the need for intensive pest control. Our study emphasizes the importance of temperature-specific management strategies, incorporating certified virus-free seed potatoes, crop rotation, insecticides, and PVY-resistant potato varieties (Dupuis et al., 2017; Krüger & vander Waals, 2020). Biological control agents, such as natural predators and parasitoids, should also be integrated into IPM programs. Additionally, understanding geographical variations in PVY transmission risk is critical for effective IPM strategies. In high-risk regions, continuous monitoring and early detection are essential, while in moderate-risk areas, targeted interventions during peak aphid activity can manage virus transmission. Implementing predictive models that incorporate climate data can forecast high-risk periods and guide control measures.

## Conclusion

Our study reveals that temperature significantly affects the life history traits and PVY-O transmission efficiency of *M. persicae*, one of the most efficient PVY-O vectors worldwide. Optimal development and highest fecundity were observed at cooler temperatures, while peak virus transmission occurred at around 20 °C. Using the ILCYM-based model, we identified regions at varying levels of PVY-O transmission risk: high in tropical and subtropical areas, moderate in temperate zones, and low in cold climates. Importantly, while our risk maps highlight areas climatically suitable for *M. persicae*, actual PVY epidemics are shaped by multiple factors, including the activity of diverse aphid vectors (particularly alates), PVY strain diversity, host susceptibility, and agronomic practices. Our findings should therefore be viewed as a model framework linking vector biology, virus transmission, and climate data, rather than as evidence that *M. persicae* alone drives PVY risk. These results underscore the need for integrated pest management (IPM) strategies tailored to specific temperature regimes, incorporating certified virus-free seed potatoes, crop rotation, insecticides, PVY-resistant varieties, and biological control agents. Looking forward, integrating multi-vector data, fluctuating temperature regimes, and field surveillance into predictive models will be critical for refining epidemiological forecasts and strengthening climate-resilient potato production systems.

Risk maps based on the Virus Transmission Index (VTI) for *M. persicae* and PVY-O have effectively identified areas susceptible to vector-borne spread, providing critical tools for guiding surveillance strategies and analysing transmission risks. These models predict that PVY-O has high establishment potential (VTI > 0.415) not only in African countries where it is already reported but also in new regions where it has not yet been introduced, highlighting the urgent need for pre-emptive monitoring and biosecurity measures.

##  Supplemental Information

10.7717/peerj.21239/supp-1 Supplemental Information 1 Supplementary Information

## Additional Information and Declarations

### Data Availability

The following information was supplied regarding data availability: The dataset and R code are available at Zenodo: Oswaldo Villena. (2026). oswaldov/Effects of temperature on life-history traits of Myzus persicae and its efficiency in transmitting potato virus Y (PVY) in potato crops (0.1.2). Zenodo. https://doi.org/10.5281/zenodo.19022905.
