# Supplementary material for: Effects of temperature on the life-history traits of Myzus persicae and its efficiency in transmitting potato virus Y (PVY) in potato crops
Source: PeerJ. 2026 Jun 4;14:e21239. doi: 10.7717/peerj.21239 (PMC13242745; doi:10.7717/peerj.21239)

**Supplemental Material:** Effects of temperature on the life-history traits of *Myzus persicae* and its efficiency in transmitting potato virus Y (PVY) in potato crops

Bonoukpoè M. Sokame^1^, Henri E.Z. Tonnang^2,3^, Heidy Gamarra^4^, Pablo Carhuapoma^4^, Jan Kreuze^4^, Leah Johnson^5^, Ali Arab^6^, Peter Armbruster^7^, Oswaldo C. Villena^8,🖂^

^1^International Centre of Insect Physiology and Ecology - icipe, Nairobi, Kenya

^2^International Institute of Tropical Agriculture – IITA, Ibadan, Nigeria

^3^School of Agricultural, Earth, and Environmental Sciences, University of KwaZulu-Natal, Pietermaritzburg 3209, South Africa

^4^The International Potato Center - CIP, Lima, Peru

^5^Department of Statistics, Virginia Tech, Blacksburg, VA 24061 USA

^6^Department of Mathematics and Statistics, Georgetown University, Washington, DC 20057 USA

^7^Department of Biology, Georgetown University, Washington, DC 20057 USA

^8^The Earth Commons Institute, Georgetown University, Washington, DC 20057 USA

Corresponding author:

Oswaldo C. Villena^8,🖂^

Email Address: oswaldo.villena@georgetown.edu

**Table S1:** Nymphal and adult development time and mortality rate for *Myzus persicae* under different temperatures using the Insect Life Cycle Modeling (ILCYM) software.

| Temp. |  |  | Nymphs | |  |  | Adult | | |  |
| --- | --- | --- | --- | --- | --- | --- | --- | --- | --- | --- |
|  | N ^A^ |  | Median dev. time | | Mortality |  | N | Median dev. time | |  |
| (°C) |  |  | (days) ^C^ | | (%) |  |  | (days) | |  |
| 10 | 100 |  | 12.83 (0.824) | | 42 |  | 50 | 11.43 (0.872) | |  |
| 15 | 100 |  | 11.27 (0.944) | | 20 |  | 79 | 11.69 (1.132) | |  |
| 20 | 100 |  | 10.11 (0.902) | | 32.2 |  | 62 | 9.59 (0.977) | |  |
| 25 | 100 |  | 10.36 (0.874) | | 22 |  | 69 | 10.69 (1.065) | |  |
| 30 | 100 |  | 11.47 (1.182) | | 64 |  | 36 | 6.06 (0.711) | |  |
|  | Model ^B^ |  | Weibull | |  |  | Weibull | | |  |
|  | *ln*(scale) |  | -2.959 (0.043)*** | |  |  | -0.9738 (0.0470)*** | | |  |
|  | Scale *δ* |  | 0.0519 (0.002)*** | |  |  | 0.378 (0.018)*** | | |  |
|  | *α = 1/ δ* |  | 19.3 (0.84)*** | |  |  | 2.64 (0.124)*** | | |  |
|  | |  | *ln* L | Deviance |  |  | *ln* L | | Deviance |  |
| Intercept only | |  | -757.6 |  |  |  | -871.6 | |  |  |
| for each Temp. | |  | -731.7 | 1469.38 |  |  | -842.9 | | 142.32 |  |
| P | |  | <0.001 | | 0.2472 |  | <0.001 | | |  |

^A^ N is the number of individuals evaluated at a given temperature.

^B^ *δ* is the scale of the selected distribution link function; the figures in () are SE of *ln*(*δ*), *δ*, and *α* (“***” indicates P < 0.001). The accumulated development frequency in relation to normalized age (time/median time) is calculated according to the selected distribution link function; for example, for the log-logistic link function: accu. *dev. freq. = 1-(1/(1+x^α^)),* where *x* is the normalized age (determined through rate summation), and *α* = *1/δ*.

^C^ Numbers in parenthesis are 95% confidence limits based on t-distribution (a heterogeneity factor, *H = deviance/df*, was included to calculate the limits). Medians followed by different letters in the same columns are significantly different (P < 0.05) according to the AFT mode

**Table S2:** Models and their parameters fitted to describe the development rate (1 per day) for immature life stages of *M. persicae* reared on potato plants.

| Life Stages | Parameter estimates of the model^A^ | | | *F* value | | *df* _1,2_ | *P* | AICc |
| --- | --- | --- | --- | --- | --- | --- | --- | --- |
|  | *T_opt_* | *T_roh_* | *r_m_* |  | |  |  |  |
| Nymph | 21.93 (±0.32)** | 17.69 (±0.8)* | 0.097 (±0.001)** | | 86.37 | 2, 2 | 0.011 | -17.1 |

Numbers in parenthesis are standard errors. Parameter values significantly different from zero are indicated by asterisks (P < 0.05 = *, P < 0.01 = **, P < 0.001 = ***).^A^ The equation of the Taylor model is:

$$r(T)=r_{m}*e^{-\frac{1}{2}\left( -\frac{\left( T-T_{opt} \right)}{T_{roh}} \right)^{2}}$$

where *r*(*T*) is the development rate at temperature *T*, then *T_opt_* , *T_roh_* and *r_m_*, are parameters of the equation.

**Table S3:** Models and their parameters fitted to describe the mortality rate for immature life stages of *M. persicae* reared on potato plants.

| Life stages | Parameter estimates of the Quadratic model^A^ | | | *F* value | *df*_1, 2_ | *P* | AICc |
| --- | --- | --- | --- | --- | --- | --- | --- |
|  | *a* | *b* | *c* |  |  |  |  |
| Nymph | 0.013 (±0.7)** | -0.49 (±0.24)** | 3.25 (±2.22)* | 3.04 | 2, 2 | 0.247 | 11.8 |

Numbers in parenthesis are standard errors. Parameter values significantly different from zero are indicated by asterisks (P < 0.05 = *, P < 0.01 = **, P < 0.001 = ***). ^A^ The equation of the Quadratic model is:

$$m\left( T \right)=a+b(T)+ c( T^{2})$$

where *m*(*T*) is the mortality at temperature *T*, then *a*, *b* and *c*, are parameters of the equation.

**Table S4:** Median oviposition time and mean fecundity for *Myzus persicae* under different temperatures

| Temp. | N |  | Median oviposition time | |  | Mean fecundity | |
| --- | --- | --- | --- | --- | --- | --- | --- |
| (°C) |  |  | (days)(±SE) | |  | (eggs/female) (±SE) | |
| 10 | 50 |  | 5.87 (0.839) | |  | 30.16 (1.919) | |
| 15 | 79 |  | 6.51 (1.116) | |  | 46.25 (2.405) | |
| 20 | 62 |  | 4.99 (0.921) | |  | 39.68 (2.084) | |
| 25 | 69 |  | 5.74 (1.036) | |  | 39.52 (2.598) | |
| 30 | 36 |  | 3.09 (0.916) | |  | 14.66 (1.107) | |
|  | Model ^B^ |  | Weibull | |  |  | |
|  | *ln*(scale) |  | -0.4391 (0.007)*** | |  |  | |
|  | Scale *δ* |  | 0.6446 (0.004)*** | |  |  | |
|  | *α* = 1/ *δ* |  | 1.5513 (0.012)*** | |  |  | |
|  | |  | *ln* L | Deviance |  |  |  |
| Intercept only | |  | -34221.6 |  |  |  |  |
| for each Temp. | |  | -33927.7 | 7182.3 |  |  |  |
| P | |  | <0.001 | |  | 0.079 | |

^A^ N is the number of individuals evaluated at a given temperature.

^B^ *δ* is the scale of the selected distribution link function; the figures in () are SE of *ln*(*δ*), *δ*, and *α* (“***” indicates P < 0.001). The accumulated development frequency in relation to normalized age (time/median time) is calculated according to the selected distribution link function; for example, for the log-logistic link function: accu. *dev. freq. = 1-(1/(1+x^α^))*, where *x* is the normalized age (determined through rate summation), and *α = 1/δ*.

**Table S5:** Models and their parameters fitted to describe adult senescence rate, total number of eggs per female, and oviposition time for *M. persicae* reared on potato plants.

| Response variable | Models ^a^ | Parameters | *F* value | df _1,2_ | *P* | AICc |
| --- | --- | --- | --- | --- | --- | --- |
|  |  | $b_{0}$ 0.139 (±0.002) ^b^ | 3.523 | 2,2 | 0.221 | 30.93 |
| Adult senescence rate | Quadratic: *s*$\left( T \right)=$  $b_{0}+b_{1}(T)+ b_{2}( T^{2})$ | $b_{1}$ -0.007 (±0.007) |  |  |  |  |
|  |  | $b_{2}$ 0.0003 (±0.001) |  |  |  |  |
|  |  |  |  |  |  |  |
|  |  |  |  |  |  |  |
| Total eggs per female | Quadratic: *t*$\left( T \right)=$  $b_{0}+b_{1}(T)+ b_{2}( T^{2})$ | $b_{0}$ -0.007 (±0.071) | 11.639 | 2,2 | 0.0791 | 0.58 |
|  |  | $b_{1}$ 0.2754 (±0.078) |  |  |  |  |
|  |  | $b_{2}$ 1.4069 (±0.724) |  |  |  |  |
|  |  |  |  |  |  |  |
| Oviposition time^-1^ | Taylor: $o\left( T \right)=$  $r_{m}*e^{-\frac{1}{2}\left( -\frac{\left( T-T_{opt} \right)}{T_{roh}} \right)^{2}}$ | $T_{opt}$ 15.109 (±3.762) | 3.436 | 2,2 | 0.225 | 24.03 |
|  |  | $T_{roh}$ 16.407 (±5.93) |  |  |  |  |
|  |  | $r_{m}$ 1.8456 (±0.127) |  |  |  |  |

a Models: Quadratic: *s(T)* is the senescence rate at temperature *T* (°C), and *b* is equation parameters. Quadratic: *t(T)* represents the fecundity function at temperature *T* (°C) and *b_1_*, *b_2_*, and *b_3_* are parameters of the equation, and *o(T)* is the inverse oviposition time where *T_opt_, T_roh_*, *and r_m_* are parameters of the equation.

b Numbers in parenthesis are standard errors.

**Table S6:** Table of results of the transmission percentage based on the number of infected potato plants per plant of potato virus Y (PVY) by *M. persicae*

| Temp. | N ^A^ |  | Average number of infected |  | Average of Number of |
| --- | --- | --- | --- | --- | --- |
| (°C) |  |  | plants from 3 replicates |  | Infected/total plant (%) (±SE) |
| 10 | 10 |  | 1.333 |  | 13.33 (±0.033) |
| 15 | 10 |  | 2.333 |  | 23.33 (±0.088) |
| 20 | 10 |  | 1.666 |  | 16.66 (±0.088) |
| 25 | 10 |  | 0.333 |  | 3.33 (±0.033) |
| 30 | 10 |  | 1.333 |  | 13.33 (±0.033) |
|  | Model ^B^ |  |  | Taylor |  |
|  | AIC |  |  | -5.182 |  |
| P | |  |  | 0.023 |  |

^A^ N is the number of plants evaluated at a given temperature.

^B^ Name of the nonlinear model.

**Table S7:** Models and their parameters fitted to describe the acquisition of potato virus Y (PVY) by *Myzus persicae* in potato plants

| Response variable | Models ^a^ | Parameters | *F* value | df _1,2_ | *P* | AICc |
| --- | --- | --- | --- | --- | --- | --- |
|  |  |  |  |  |  |  |
| Adult senescence rate |  | $T_{opt}$ 17.435 (±0.688)^b^ | 5.871 | 2,9 | 0.023 | -5.182 |
|  | Taylor: $m\left( T \right)=$  $1-rm*e^{\left( -\frac{1}{2}\left( -\frac{\left( T-T_{opt} \right)}{T_{roh}} \right)^{2} \right)}$ | $T_{roh}$ 7.177 (±1.172) |  |  |  |  |
|  |  | $r_{m}$ 0.2077 (±0.016) |  |  |  |  |
|  |  |  |  |  |  |  |

a Models: Taylor: *m(T)* is the transmission rate function, where *T_opt_*, *T_roh_*, and *r_m_* are parameters of the equation.

b Numbers in parenthesis are standard errors.

**Figure S1:** Diagram showing the procedure of virus transmission experiments of potato virus Y (PVY) by *Myzus persicae* under constant temperatures (12°C,15°C, 20°C, 25°C).


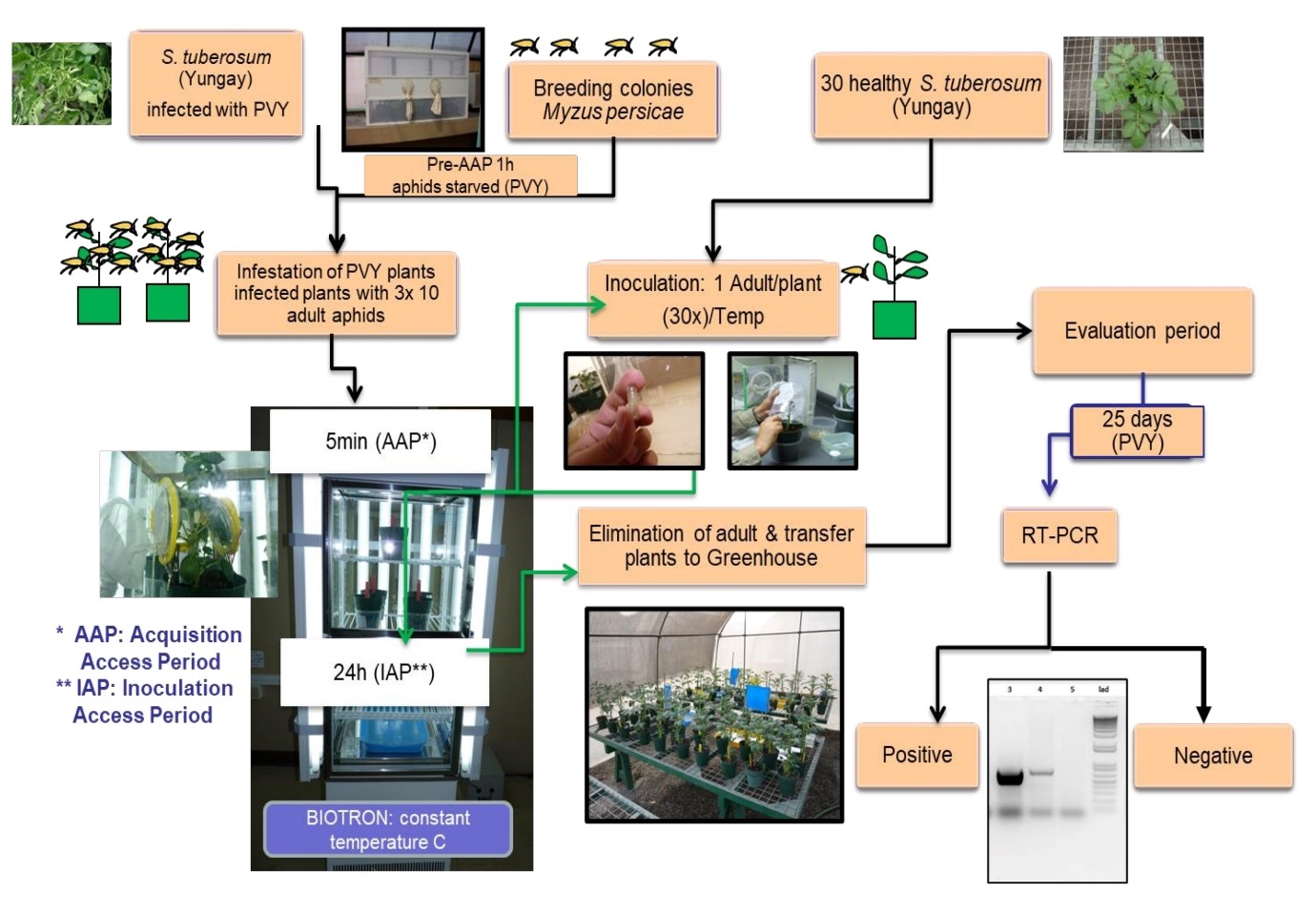

Supplement: Supplemental Information 1 [file peerj-14-21239-s001.docx]
